# Defect Engineering in Silver-Based Bimetallic Semiconductors: Recent Advances and Future Perspective

**DOI:** 10.1021/acsomega.5c00524

**Published:** 2025-05-28

**Authors:** Marcelo Assis, Ana Claudia Muniz Rennó, Juan Andrés, Elson Longo

**Affiliations:** 1 Department of Biosciences, 28105 Federal University of São Paulo (UNIFESP), Santos, SP 11015-020, Brazil; 2 Department of Analytical and Physical Chemistry, University Jaume I (UJI), Castelló 12071, Spain; 3 CDMF, LIEC, 67828 Universidade Federal de São Carlos (UFSCar), São Carlos, SP 13565-905, Brazil

## Abstract

Since the mid-20th century, the interaction between light and inorganic semiconductors plays not only a key role in numerous fascinating phenomena but also provides the physical foundations for the development of many modern technologies focused on health, environmental, and energy solutions. Among these materials, silver-based bimetallic semiconductors have garnered attention due to their enhanced functional properties, which are controlled by the presence and distribution of structural and electronic defects. These defects directly impact key physicochemical properties, making them essential for the development of materials with improved functionalities. Modifying synthetic and postsynthetic parameters is crucial for controlling the type, density, and distribution of these defects in materials. However, achieving precise control of these defects remains a challenge and requires a deeper understanding of the relationship between synthetic conditions and defect formation. This work provides a comprehensive review of how modifications in synthesis methods influence material properties, with a particular focus on understanding their impact on material defects. Specifically, this study examines silver-based bimetallic semiconductors, including Ag_2_WO_4_, Ag_2_MoO_4_, and Ag_2_CrO_4_. Additionally, strategies involving advanced defect characterization techniques such as photoluminescence spectroscopy (PL), X-ray photoelectron spectroscopy (XPS), electron paramagnetic resonance (EPR), and positron annihilation lifetime spectroscopy (PALS) are discussed, as these methods are gaining prominence in defect analysis. By exploring the interplay between synthetic control and its impact on defects in these materials, this study highlights the critical role of defect engineering in advancing the application potential of silver-based bimetallic semiconductors.

## Graphical abstract

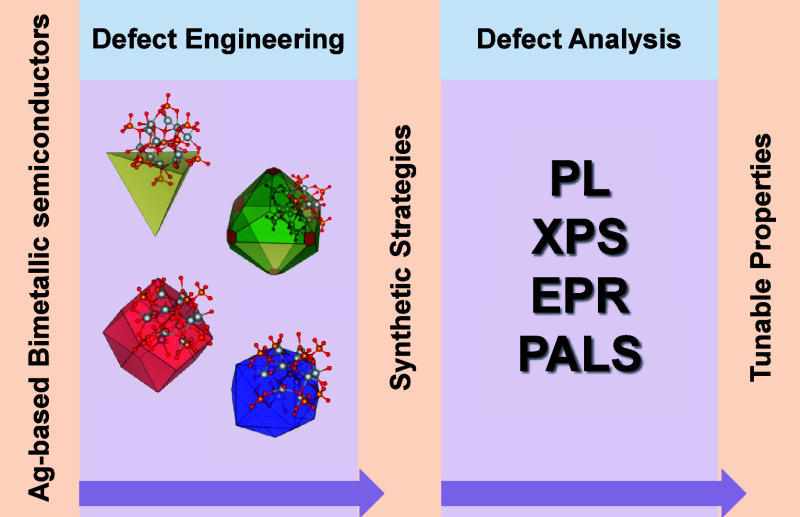

## Introduction

In recent years, inorganic semiconductors have gained increased prominence in academia and industry by their potential to overcome the challenges of availability, high-cost processing, and toxicity, which have been insurmountable for the development of new technologies that enhance quality of life on a global scale. According to the Semiconductor Industry Association (SIA), global semiconductor sales reached 527 billion dollars in the second half of 2023, and future projections indicate that this figure could triple within the next decade.^1^ This growing demand is expected to stimulate investments in this field, not only for the development of traditional semiconductors used in electronic devices but also for the advancement of cutting-edge technologies that can leverage the unique properties of these materials. Historically, the center of semiconductor investments has centered around conventional electronic applications, such as components in everyday devices. However, as science and societal needs continue to evolve, emerging fields - such as catalysts and biomaterials - are gaining increased prominence. This shift reflects the inherent versatility of these materials to be an indispensable component in a wide range of sectors, from healthcare to environmental protection, and enabling them to address increasingly complex technological and social challenges.

Classically, a semiconductor is defined as a material whose electronic structure features a moderate band gap between the valence band (VB), comprising orbitals filled with electrons strongly bound to the atoms, and the conduction band (CB), made up of empty or partially filled orbitals where electrons (e^–^) can move freely, thus enabling electrical conductivity.^2^ Unlike conductors, which have overlapping VB and CB, and insulators, which have very wide band gaps, semiconductors allow some electrons to be excited from the VB to the CB through thermal, optical, or electrical stimulation for example.^3^ This process generates charge carriers that are highly reactive, paving the way for numerous technological applications. However, the classical definition of semiconductors, typically based on bulk materials, becomes more complex when particle size is reduced and quantum effects come into play. Under these conditions, the semiconductor’s surface takes on a crucial role.^4^ Each crystallographic plane may exhibit slightly different band gaps due to specific atomic arrangements at the surface and the presence of under-coordinated atomic sites, called clusters.^5^ They generate regions of varying electron density, both positive and negative, which function similarly to charge carriers in conventional bulk semiconductors. As a result, the morphology and particle size play a critical role in determining the electronic behavior and the overall activity of the semiconductor.^6^ These clusters are closely linked to atomic vacancies present on different crystal surfaces, which exhibit distinct local metal coordination environments and unsaturated bonds. Consequently, they differ in several aspects, including their local electric field effects and electron confinement capabilities.^7^

Defects are general perturbations of the periodic atomic arrangements, which inevitably exist in semiconductor materials. i.e. they are ubiquitous and have a pronounced effect on their physical and chemical properties and thus open new opportunities for obtaining efficient defected materials As it was noted: “*Even if there is one atom vacancy in one hundred million host atoms, the electronic structure of the material will change intensely*”.^8^ Oxygen vacancies being intrinsic defects commonly found on the surface of real semiconductors, even in infinitesimal concentration, are found to play a more decisive role in determining the kinetics, energetics, and reactive mechanisms.^9^ Specifically, introducing defects into these materials induces the formation of intermediate electronic states within the forbidden region of the band gap.^10^ Such defects affect electronic transitions between the atomic clusters that form the material, allowing for fine-tuning of its electronic structure and charge distribution. Therefore, defect engineering is recognized as an effective route to obtaining highly active materials by tuning the atom coordination number and electronic structure, optical, charge separation, and surface properties, and therefore to improve their activity, making them suitable for a wide range of functional applications.^5^

It is important to note that processes such as the formation of surface clusters with different electronic densities and the creation of prepopulated intermediate electronic states occur in all semiconductors, including established examples like TiO_2_, ZnO, WO_3_, CeO_2_, Fe_2_O_3_, and Fe_3_O_4_. Ag-based bimetallic semiconductors, such as Ag_2_WO_4_, Ag_2_CrO_4_, and Ag_2_MoO_4_, are particularly interesting because they introduce additional complexity to the electronic structure by combining the unique properties of Ag and metal cations with oxygen-based lattices.^11^ This combination not only enhances their electronic and optical properties but also promotes superior performance in applications like photocatalysis, antimicrobial activity, and energy conversion, making them ideal candidates for advanced technological solutions.

In this context, Ag_2_WO_4_, Ag_2_CrO_4_, and Ag_2_MoO_4_ semiconductors have attracted growing interest due to their well-defined crystal structures and their capacity for extensive physicochemical tailoring. Among them, Ag_2_WO_4_ stands out not only for its polymorphic diversity but also for its remarkable responsiveness to external excitation, including electron beams and laser irradiation, which can induce the formation of metallic Ag nanoparticles directly on the material’s surface, as highlighted by Gouveia et al.^12^ This phenomenon, also observed in Ag_2_CrO_4_ and Ag_2_MoO_4_, reflects the intrinsic photo- and electro-sensitivity of these Ag-based semiconductors, allowing their surface reactivity to be tuned without altering the bulk crystalline framework.^13–15^ Thanks to its broad-spectrum activity and high chemical reactivity, Ag_2_WO_4_ has been successfully employed in a range of applications, including photocatalytic degradation of organic pollutants, antimicrobial coatings, and as a component in heterojunction systems for environmental and energy-related technologies.^16^ Ag_2_MoO_4_ has demonstrated strong potential in redox-based catalysis, including Fenton-like and ozonation processes, owing to its efficient electron transfer behavior and chemical stability.^17^ Meanwhile, Ag_2_CrO_4_ stands out for its high absorption in the visible spectrum and pronounced photoactivity, making it particularly effective for solar-driven degradation of pollutants.^18^ Together, these materials offer tunable band structures, controlled morphologies, and dynamic surface states, key attributes that support efficient charge separation, robust reactive oxygen species (ROS) generation, and high operational stability. As such, their combined structural, electronic, and functional advantages position them as compelling platforms for innovations in photocatalysis, environmental remediation, renewable energy, and biomedical applications.

To further enhance their functional properties, defect engineering has emerged as a powerful strategy to manipulate the electronic and surface characteristics of these materials. Introducing and controlling defects not only improves catalytic activity but also plays a key role in modifying surface reactivity, particularly by facilitating the adsorption of reactants and the generation of active sites. In this regard, the incorporation of different metal cations within a single crystalline lattice promotes distinctive electronic interactions with oxygen anions, leading to the formation of intermediate states within the band gap.^19,20^ As a result, it becomes possible to finely tune energy levels and improve charge carrier mobility. Additionally, the intrinsic structural complexity of these materials, expressed through their diverse atomic clusters and crystallographic terminations, can significantly influence their surface dynamics, even under dark conditions.^21^ This variety of clusters types is not merely a structural feature, but a key factor in enabling the formation of a wide variety of structural defects, such as vacancies, distortions, and nonstoichiometric sites.^22^ These defects, in turn, directly modulate the material’s reactivity by influencing parameters like charge carrier separation, adsorption capacity, and the generation of reactive oxygen species. As a result, Ag-based bimetallic semiconductors become highly effective in processes that rely on efficient charge transport, reactive species production, or selective interaction with electromagnetic radiation, reinforcing their growing relevance in catalysis, sensing technologies, energy conversion systems, and functional biomaterials.

Building upon this foundation, the present study investigates how different synthetic strategies for obtaining Ag-based bimetallic semiconductors, Ag_2_WO_4_, Ag_2_CrO_4_, and Ag_2_MoO_4_, can modulate defect density, chemical composition, and spatial distribution within the material matrix. A range of complementary characterization techniques are explored to analyze these defect structures and their impact on functionality. By correlating synthetic parameters with defect-related behavior, this work provides valuable insights for the rational design of efficient and multifunctional Ag-based semiconductors, offering a roadmap for future developments in this rapidly evolving field. Unlike previous works, this review provides a comprehensive overview of pristine Ag-based materials, highlighting their structural and functional versatility, current synthetic advancements, and unresolved challenges, thereby extending the existing knowledge in the field.

## Structural and Electronic Features

At first glance the primary difference among Ag_2_WO_4_, Ag_2_CrO_4_, and Ag_2_MoO_4_ materials lies in the choice of lattice formers, while Ag consistently serves as the lattice modifier across all compounds (see Figure 1A).

**Figure 1:**
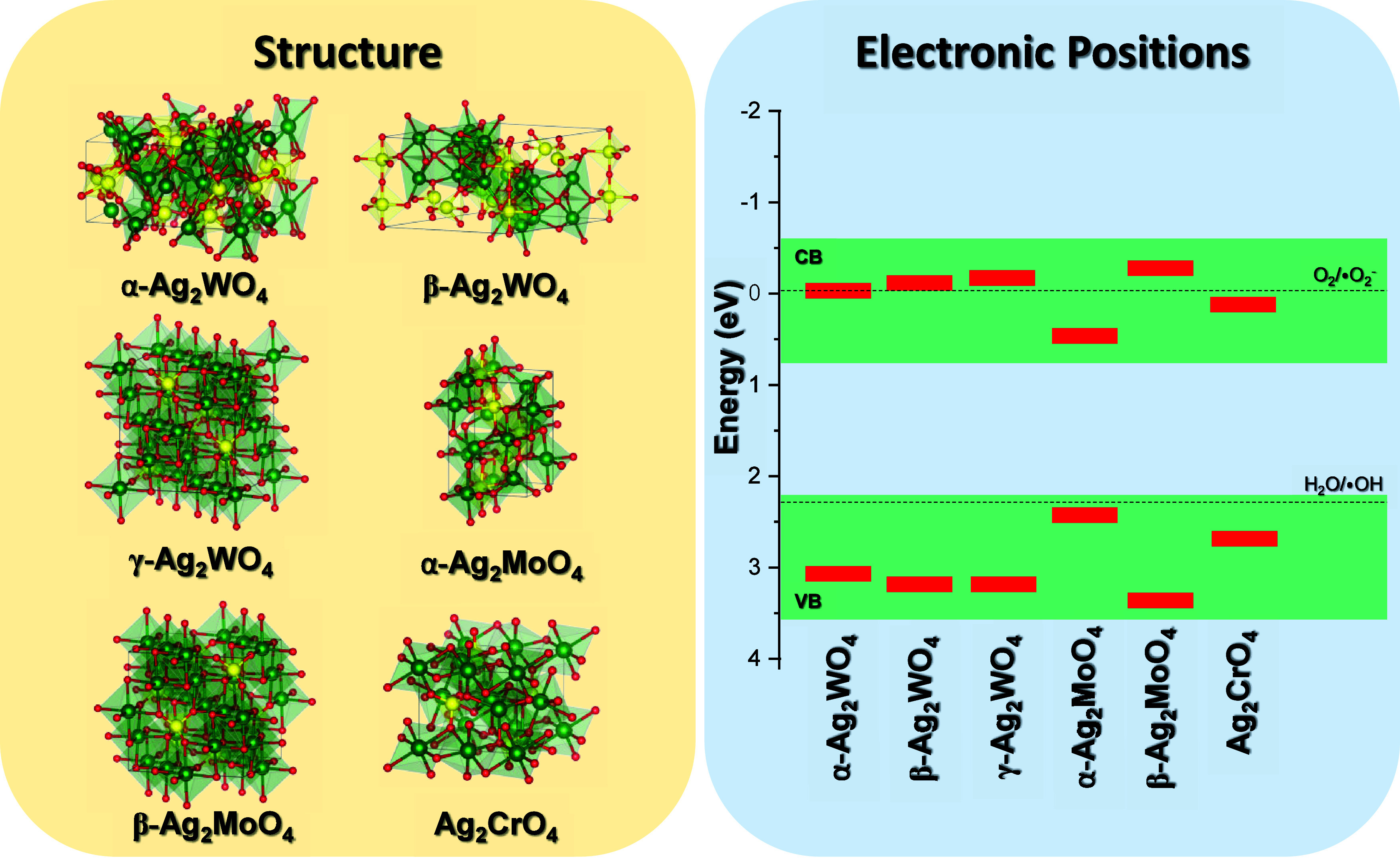
(A) Structure and (B) electronic positions of VB and CB of silver-based bimetallic semiconductors.

Among these materials, Ag_2_WO_4_ has attracted significant attention due to its advanced antimicrobial properties, which often outperform conventional Ag nanoparticles.^12^ This material exhibits three polymorphs: the thermodynamically stable α-Ag_2_WO_4_, the metastable β-Ag_2_WO_4_, and the least stable γ-Ag_2_WO_4_.^23^ The α-Ag_2_WO_4_ polymorph has an orthorhombic structure with the space group *Pn2n* and a remarkably complex arrangement at the cluster level. It is composed of three types of distorted tetrahedral [WO_6_] clusters and six distinct Ag clusters, including two [AgO_7_], one [AgO_6_], two [AgO_4_], and one [AgO_2_].^24^ This structural complexity allows for a wide variety of defects to form in both the bulk and surface of the material. In contrast, the β-Ag_2_WO_4_ polymorph has a hexagonal structure with the space group *P63/m*, featuring two types of W clusters ([WO_4_] and [WO_5_]) and two types of Ag clusters ([AgO_5_] and [AgO_6_]).^25^ Lastly, the γ-Ag_2_WO_4_ polymorph, which is the least stable, adopts a cubic spinel structure with the space group *Fd3̅m*. Its simpler architecture consists of [WO_4_] and [AgO_6_] clusters.^26^

Similarly, Ag_2_MoO_4_ exists in two polymorphs: the thermodynamically stable β-Ag_2_MoO_4_ and the metastable α-Ag_2_MoO_4_. The β-Ag_2_MoO_4_ polymorph has a cubic spinel structure, identical to γ-Ag_2_MoO_4_, with the space group *Fd3̅m*. It is composed of [MoO_4_] and [AgO_6_] clusters.^27^ The α-Ag_2_MoO_4_ polymorph, on the other hand, adopts an inverse tetragonal spinel structure with the space group *P4122*.^17^ This structure is more complex, owing to its different coordination number, and consists of [MoO_6_] and [AgO_6_] clusters.^28^ In contrast to the aforementioned materials, Ag_2_CrO_4_ do not exhibit polymorphism. The structure of Ag_2_CrO_4_ is orthorhombic, with the space group *Pnma*, and is composed of [AgO_4_], [AgO_6_], and [CrO_4_] clusters.^13^ These structural differences reflect their suitability for specific applications, particularly in photocatalysis.

From an electronic perspective, most of these materials are classified as p-type semiconductors, meaning that holes are the predominant charge carriers.^22^ The exception is α- Ag_2_MoO_4_, which is an n-type semiconductor characterized by electrons as the primary charge carriers. This distinction arises from the higher density of states in the CB of α-Ag_2_MoO_4_. The VB in these materials is primarily formed by contributions from Ag and O atoms, while the lattice formers play a more significant role in shaping the CB. The band gap (*E*_gap_) of these materials varies widely, reflecting their diverse electronic properties. For Ag_2_WO_4_ polymorphs, the *E*_gap_ ranges from 3.0 to 3.3 eV.^23^ In the case of Ag_2_MoO_4_, the stable β-phase has the highest *E*_gap_, between 3.4 and 3.6 eV,^29^ while the metastable α-phase has the lowest *E*_gap_, ranging from 1.2 to 1.4 eV, making it particularly suitable for visible light applications.^22^ Ag_2_CrO_4_ has band gap of 1.8 eV, which are ideal for photocatalytic processes as they absorb light in the visible region of the electromagnetic spectrum.^30^ The positions of the VB and CB are critical for predicting the photocatalytic efficiency of these materials, especially their ability to generate ROS (Figure 1B). Theoretical predictions indicate that Ag_2_CrO_4_ has CB levels below the redox potential of the O_2_/•O_2_^–^ pair (0.05 eV), while α-Ag_2_MoO_4_ uniquely exhibits both a CB below the O_2_/•O_2_^–^ redox potential and a VB above the H_2_O/•OH redox potential (2.30 eV).^22^ These insights guide the development of advanced photocatalytic systems for environmental remediation, highlighting the importance of managing ROS production for efficient pollutant removal.

## Defects in Semiconductors

In a classical description of a semiconductor, one might ideally expect a perfectly ordered crystalline lattice, with every atom occupying its intended site. However, structural and electronic imperfections - commonly referred to as defects - inevitably exist in semiconductor materials along the crystal growth process.^31,32^ As mentioned above, these defects can profoundly influence a semiconductor’s electrical, optical, mechanical, and chemical properties.^33–35^ While the traditional understanding of semiconductors often focuses on bulk-scale behavior, advances in nanotechnology and surface science have unveiled a complex variety of surface defects that open pathways to innovative applications and tailored functionalities (Figure 2).

**Figure 2:**
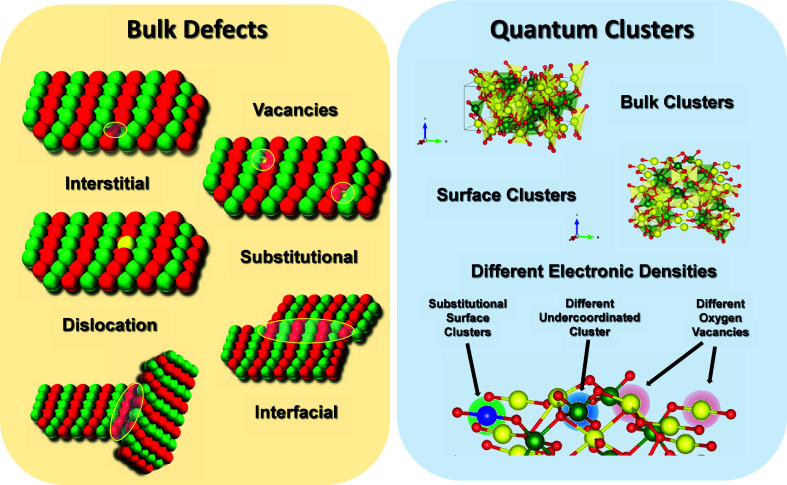
(A) Classical and (B) quantum surface defects.

When considering a bulk semiconductor in its most straightforward form, defects typically manifest as point defects (vacancies, interstitials, or substitutional atoms), line defects (dislocations), or surface/interface defects (grain boundaries and phase boundaries).^36^ Point defects often emerge when an atom is missing, occupying an incorrect location, or substituted by a different species, and each of these scenarios can introduce localized states in the band structure. Dislocations arise along lines in the crystal where the periodic atomic arrangement is disrupted, creating areas of stress that can scatter charge carriers. Surface or interface defects become relevant when grains of different orientations meet, or when a secondary phase merges with the primary phase.

These defects can profoundly influence a semiconductor’s electrical, optical, mechanical, and chemical properties.^33–35^ While the traditional understanding of semiconductors often focuses on bulk-scale behavior, advances in nanotechnology and surface science have unveiled a complex variety of quantum and surface defects and vacancies which are responsible of a plethora of quantum effects, making them suitable for multidomain applications and innovative functionalities (Figure 2). Different research groups have published works aimed at elucidating the role of defects and vacancies in modulating the electronic structure, optical, and surface-active sites.^37,38^

As the focus shifts from the bulk structure to the material’s surfaces - especially at the nanoscale -the concept of defects broadens to include what can be termed “quantum” or “surface” defects.^39,40^ In semiconductors with a wide array of structural clusters, the notion of quantum clusters as a type of quantum defect becomes critically important.^21^ These quantum clusters emerge from local alterations in electronic density and may have multiple origins. They can be intrinsic - such as undercoordinated surface clusters - or induced through synthetic modifications that increase the prevalence of these undercoordinated sites.^41^ Additionally, the incorporation of foreign elements into the semiconductor’s structure can trigger a cascade effect, adjusting local electronic density to compensate for electron deficits or surpluses. This expanded concept of quantum clusters also helps clarify how charge-carrier pairs (electrons and holes) are formed and behave within a semiconductor. By creating discrete electronic states in the band gap that are mirrored in different surface electronic densities, these clusters can influence the dynamics of electron–hole generation, trapping, and recombination. Whether they bolster beneficial interactions (e.g., enhanced adsorption of reactants) or promote unfavorable electron–hole recombination, quantum clusters strongly affect the material’s overall functionality and performance.

Vacancies, whether metallic or oxygen-related, play a crucial role in determining the structural, electronic, and functional properties of semiconductors.^42,43^ Metallic vacancies, often associated with cationic sites, can disrupt lattice symmetry and create localized electronic states, altering charge distribution and mobility. Similarly, oxygen vacancies, among the most studied, introduce intermediate energy levels within the bandgap that directly influence optical and electronic behaviors, such as photoluminescence, conductivity, and catalytic activity.^44,45^ These defects, whether present in bulk or concentrated at the surface within quantum clusters, serve as key sites for chemical reactions, adsorption, or charge trapping, making them integral to the performance of semiconductor-based technologies. Oxygen vacancies, in particular, are critical for tailoring semiconductor functionality.^46^ They often determine the relationship between the degree of structural order/disorder and electronic dynamics, acting as centers for electron trapping and recombination. In quantum clusters, undercoordinated clusters with different oxygen vacancies modify the local surface electronic density, leading to changes in bandgap energies and directly impacting the surface properties of the material. This alteration can be observed in various applications, such as the generation of ROS, where oxygen vacancies can enhance their production, or in the surface interactions of these materials with other molecules, as they can amplify surface reactivity.

To rationally exploit defect production in materials, such as vacancies, it is crucial to use appropriate characterization techniques, in addition to maintaining rigorous synthetic control, as even small modifications can be critical to defect formation. Strict synthetic control directly influences the degree of order/disorder in these materials and, consequently, their defects, enabling fine-tuning of material performance. Synthetic and postsynthetic approaches that can effectively modify nature and distribution of these material defects will be highlighted and analyzed in detail in the next section. Equally important to synthetic control is the use of advanced characterization techniques to analyze and, where possible, quantify these defects, as structure–property correlations are essential for the rational development of materials. Techniques such as photoluminescence spectroscopy (PL), X-ray photoelectron spectroscopy (XPS), electron paramagnetic resonance (EPR), and positron annihilation lifetime spectroscopy (PALS) have gained prominence for identifying the types, densities, and spatial distributions of surface defects, particularly those related to vacancies. Like synthetic strategies, these techniques will also be discussed in subsequent sections.

## From Defects to Applications

Rather than being seen merely as imperfections, structural defects in Ag-based semiconductors are now recognized as critical elements for tuning the activity of advanced materials. When rationally introduced, these defects act as functional levers that modulate surface chemistry, charge transport, and overall reactivity, parameters essential for applications such as photocatalysis, gas sensing, and antimicrobial action. Importantly, the presence of defects induces short-range structural distortions, which are directly reflected in the local electronic behavior, both in the bulk and at the surface. These alterations modify the dynamics of charge carrier recombination, typically by introducing new defect states within the band gap (between VB and CB) that act to slow down recombination processes (Figure 3). Depending on their energetic position, these defect states can be classified as shallow, located near the band edges, or deep, positioned closer to the center of the band gap. In either case, they fundamentally reshape the local electronic structure, thereby influencing the material’s overall functional performance.

**Figure 3:**
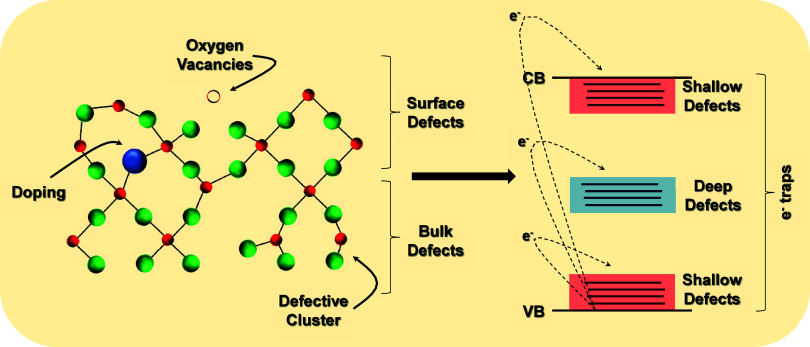
Schematic representation of how structural defects induce short-range distortions that impact local electronic properties in Ag-based semiconductors.

In photocatalytic systems, controlled introduction of oxygen vacancies can reduce the bandgap of the semiconductor, extending their photoresponse into the visible region.^34,47^ These vacancies not only facilitate light harvesting but also promote the separation of photogenerated charge carriers, lowering recombination losses.^48^ More importantly, they create active sites where redox reactions can occur more efficiently, fundamental for pollutant degradation. Similar principles apply to gas sensors and antimicrobial systems, where surface activation and reactive species generation are equally important, albeit through different mechanisms.

In antimicrobial contexts, the presence of surface defects, particularly those that involve under-coordinated Ag atoms, supports the release of Ag^+^ ions and enhances the production of ROS, expanding their full potential and enhancing their antimicrobial performance.^11^ Unlike conventional photocatalysis, where light is essential to initiate charge separation, defect-rich Ag-based semiconductors can generate ROS even in the absence of ligth.^49^ This occurs because certain defect states host pre-excited or loosely bound electrons capable of engaging in redox processes at the surface.^50^ Although the ROS generation is less intense in the dark, exposure to light significantly amplifies this activity by promoting additional charge excitation and boosting overall reactivity. In gas sensors, similar surface defects act as molecular recognition sites, where adsorbed analytes modulate local electronic properties and conductivity through specific interactions.

Doping strategies further enhance the utility of defect engineering. Incorporating heteroatoms can lead to lattice distortions that favor defect formation, such as the substitution of oxygen by nonmetals or the generation of cationic vacancies to balance charge.^51^ This approach simultaneously tunes light absorption, electronic conductivity, and surface polarity, yielding improved performance across different applications. Co-doping using two or more dopants can offer synergistic effects by balancing defect formation energy with band structure optimization.

The key mechanisms behind the performance of Ag-based semiconductors, such as ROS generation, surface charge modulation, and molecular interactions, are fundamentally linked and span their main applications. Whether employed in pollutant degradation, gas sensing, or antimicrobial activity, their functionality is inherently driven by surface-mediated processes. By precisely tailoring surface defect chemistry, it becomes possible to exploit a unifying operational principle across these diverse fields. As scientific insight advances, the development of defect-engineered materials evolves toward a defect-by-design approach, in which reactivity, selectivity, and long-term stability are deliberately integrated into the crystal structure from the very beginning.

## Synthetic Strategies for Defect Control: Defect Engineering

### Synthetic Parameters

Controlling and even inducing defects intentionally has become a key strategy for optimizing semiconductor functionality. Several synthetic and postsynthetic approaches facilitate this level of control. For instance, doping (the purposeful introduction of foreign atoms at low concentrations) can alter the density of point defects and shift the Fermi level, enhancing conductivity or manipulating light absorption.^52^ Thermal treatments in specific atmospheres (oxidizing, reducing, or inert) can trigger the formation or healing of vacancies.^53^ Reducing the particle size from bulk to nano increases surface area, thus magnifying the importance of surface and quantum defects.^54^ Adjusting the stoichiometry–such as subtly changing the ratio of metal ions to oxygen–can foster oxygen vacancies, which in turn strongly affect the electronic behavior.^55^ Physical processes like electron/light irradiation or plasma treatments can also create or repair defects in targeted regions, offering further avenues for fine-tuning the material’s properties.^56^ Understanding the nature and control of these defects is vital for designing semiconductors that meet the specific demands of emerging technologies (see Figure 4).

**Figure 4:**
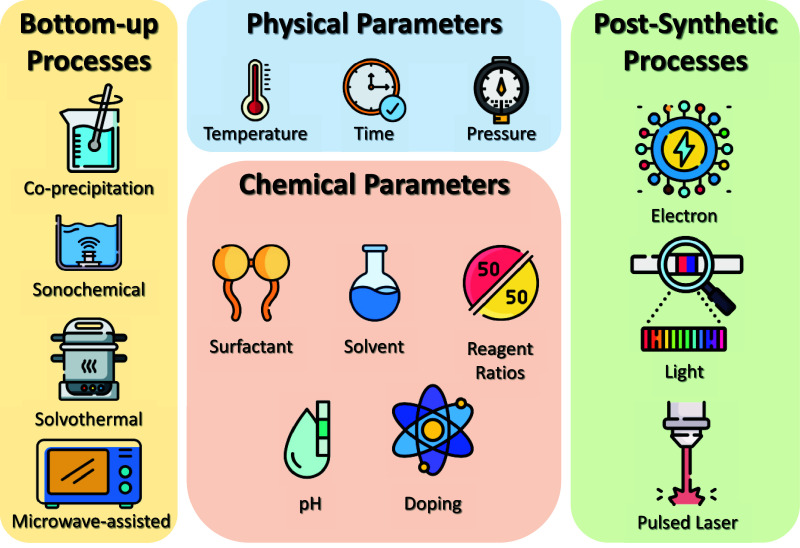
Synthetic parameters for the synthesis of silver-based bimetallic semiconductors.

These conditions profoundly impact material defects and, consequently, surface engineering outcomes. Structural surface defects, modulated by factors such as agitation, microwave power, or ultrasound intensity, create surfaces with tailored chemical and physical properties. For instance, stabilizing surfaces with quantum clusters offers distinct active sites for chemical reactions, modifying material behavior. Additionally, controlling the degree of order and disorder–reflected in the type and density of defects–affects electronic properties such as charge mobility and optical response, enabling precise tuning of the functional characteristics of semiconductors.

Ag-based bimetallic semiconductors are commonly synthesized through bottom-up processes, offering remarkable flexibility in adjusting reaction parameters to fine-tune material properties (see Table 1). Techniques such as coprecipitation, solvothermal, microwave-assisted, and sonochemical methods are widely utilized, each providing unique advantages for controlling defect density and morphology.^57^ For example, in coprecipitation methods, the mode of reagent addition (e.g., dripping, pouring, or injection) significantly influences the nucleation rate and crystal growth, thereby modulating the structural order or disorder of the synthesized materials. Our research group successfully altered the typical hexagonal rod-like morphology of α-Ag_2_WO_4_ into parallelepipeds by modifying the coprecipitation process through the injection of AgNO_3_ in a solution of DMSO containing the former lattice.^58^ Similarly, Liu et al. synthesized one-dimensional α-Ag_2_WO_4_ nanowires by introducing AgNO_3_ during a coprecipitation process, followed by microwave-assisted hydrothermal treatment and *in situ* nucleation of Ag nanoparticles.^59^ The resulting Ag/Ag_2_WO_4_ composites exhibited enhanced photocatalytic activity, which was attributed to a combination of factors: the surface plasmon resonance (SPR) effect of Ag, bandgap narrowing, and the formation of a Schottky barrier at the Ag/semiconductor interface. Together, these mechanisms contribute to improved light absorption across a broader spectral range and more efficient generation and separation of photogenerated charge carriers. We demonstrated that the morphology of Ag_2_CrO_4_ could be manipulated by adjusting the rate of lattice modifier addition during coprecipitation, transitioning from rapid direct addition (25 mL/s) to a controlled dripping process (10 mL/min).^60^ This modulation not only influenced particle size and surface structure but also played a decisive role in the type and distribution of structural defects, particularly oxygen vacancies and shallow trap states, which in turn directly impacted the material’s PL behavior and electronic properties.

**Table 1:** Summary of Different Synthesis Methods for Ag-Based Semiconductors^a^

material	synthetic method	solvent	temperature (°C)	additive	morphology	defect analysis	application	ref
β-Ag_2_MoO_4_	coprecipitation	water	90		polyhedral-like		photocatalysis of RhB	^29^
	sonochemical	water	45		potatoes-like			
	hot solution ion injection with fast cooling	water	90		irregular shape			
	conventional hydrothermal	water	90		irregular polyhedrons			
α-Ag_2_WO_4_	coprecipitation with fast injection	water and DMSO	120		hexagonal rod-like			^58^
α-Ag_2_WO_4_	conventional hydrothermal	water	150		nanowires	XPS	photocatalysis of MB	^59^
Ag_2_CrO_4_	coprecipitation	water	90		polyhedral-like	PL		^60^
α-Ag_2_WO_4_	sonochemical followed by conventional hydrothermal	water	140		hexagonal rod-like		antimicrobial	^61^
Ag_2_CrO_4_	microemulsion	cyclohexane/n-hexanol	RT	Triton X-100	spheres		photocatalysis of MB	^62^
	coprecipitation	water	RT		irregular shape			
	conventional hydrothermal	water	160		irregular shape			
α-Ag_2_WO_4_	coprecipitation	water	10–90	PVP	hexagonal rod-like and parallelepiped-like	XPS	photocatalysis of MB and MO	^63^
α-Ag_2_WO_4_	coprecipitation	water	30–70	PVP	hexagonal rod-like and parallelepiped-like	XPS	antimicrobial	^64^
α-Ag_2_WO_4_	microwave hydrothermal	water	100–160	SDS	hexagonal rod-like		antimicrobial and photocatalysis of RhB and Rh6G	^65^
β-Ag_2_MoO_4_	conventional hydrothermal	water	100–160		potatoes-like and coral-like		photocatalysis of RhB	^66^
β-Ag_2_MoO_4_	coprecipitation	water	450–525	NaOH	potatoes-like	XPS	NH_3_ gas sensor	^67^
α-Ag_2_WO_4_	microwave hydrothermal	water	140		hexagonal rod-like	PL	antimicrobial and photocatalysis of RhB	^68^
Ag_2_CrO_4_	conventional hydrothermal	water	350	NaOH	irregular shape		photocatalysis of MB and MO	^69^
β-Ag_2_MoO_4_	coprecipitation	DEG or DMSO/H_2_O	0–80		spheres, truncated octahedra, cubes, cuboctahedra		photocatalysis of RhB	^70^
α-, β-, and γ-Ag_2_WO_4_	coprecipitation	water	25		hexagonal rod-like, rod-like, and Polyhedral-like	PL and XPS	antimicrobial and photocatalysis of RhB	^23^
α-Ag_2_WO_4_	coprecipitation	water	90	SDS	hexagonal rod-like and cubes	PL	photocatalysis of RhB	^71^
α-Ag_2_WO_4_	sonochemical	water	25	citric acid, tartaric acid, and benzoic acid	rod-like	PL and XPS	photocatalysis of RhB	^72^
β-Ag_2_MoO_4_	coprecipitation	PMAA	80		hollow spheres		photocatalysis of MO	^73^
γ-Ag_2_WO_4_	coprecipitation	water	RT	PVP	rod-like and Polyhedral-like		photocatalysis of mb	^74^
β-Ag_2_MoO_4_	microwave hydrothermal	water	180	PVP	cube	PL and XPS	photocatalysis of RhB	^75^
α- and β-Ag_2_MoO_4_	coprecipitation	water		PVP	butterfly like		photocatalysis of MB	^76^
β-Ag_2_MoO_4_	microwave hydrothermal	water	120–150	NH_4_OH	polyhedral-like and octahedral rod	PL		^77^
Ag_2_CrO_4_	coprecipitation	water	30–90	NH_4_OH	polyhedral-like	PL	antimicrobial and Photocatalysis of RhB	^78^
β-Ag_2_MoO_4_	microwave hydrothermal	water	150	PVP	coral-like		antimicrobial and Photocatalysis of RhB	^79^
	microwave hydrothermal	ethanol			cube			
	conventional hydrothermal	water		KOH	coral-like			
α-Ag_2_WO_4_	coprecipitation	water or ethanol	90	NH_4_OH	hexagonal rod-like	PL	antimicrobial	^80^
β-Ag_2_MoO_4_	coprecipitation	water or ethanol	90	NH_4_OH	polyhedral-like	PL	antimicrobial	^81^
β-Ag_2_MoO_4_	coprecipitation	water or ethanol	90	NH_4_OH	polyhedral-like	XPS	antimicrobial	^82^
β-Ag_2_MoO_4_	coprecipitation	water, methanol, ethanol, propanol, or 1-butanol	60		irregular shape			^83^
α- and β-Ag_2_WO_4_	coprecipitation	water	60		rod-like	PL	photocatalysis of reactive orange 86	^84^
β-Ag_2_MoO_4_	coprecipitation	water	RT	PVP	cubes, octahedral, and rods			^85^
Ag_2_CrO_4_	microwave hydrothermal	water	180		irregular shape		photocatalysis of PCP-Na	^86^
α-Ag_2_WO_4_	sonochemical	water	70	citric acid	nano rod		antimicrobial	^87^
α-Ag_2_WO_4_	coprecipitation	water	70		hexagonal rod-like		cytotoxicity	^57^
	coprecipitation			SDS	cube			
	sonochemical			citric acid	nano rod			

a RT = room temperature; RhB = rhodamine B; MO = methyl orange; MB = methylene blue.

In solvothermal and microwave-assisted synthesis, parameters such as applied power, agitation, and reaction time significantly impact defect density and distribution. Microwave-assisted methods, for instance, provide rapid and uniform heating, promoting homogeneous structures through repeated dissolution and recrystallization processes.^88^ Lower microwave power conditions, however, may result in higher defect densities. Sonochemical methods also offer precise defect engineering capabilities, as the intensity of ultrasonic power determines the cavitation effects that create specific microstructural defects under localized high-energy conditions. Nobre et al. synthesized α-Ag_2_WO_4_ using the sonochemical method alone and in combination with a conventional hydrothermal process.^61^ They observed that combining these methods induced morphological changes, enhancing the material’s antimicrobial efficiency. MIC values confirmed the higher susceptibility of *E. coli*, with values as low as 31.25 μg/mL, and 250 μg/mL for MRSA, both obtained from the sample synthesized by the sonochemical method followed by 12 h of conventional hydrothermal treatment. Similarly, Lopes et al. studied the synthesis of β-Ag_2_MoO_4_ using coprecipitation, sonochemical, hot-solution ion injection with rapid cooling, and conventional hydrothermal methods.^29^ They found that each synthesis route led to distinct morphologies and stabilized surfaces, affecting the types of surface quantum clusters formed. Notably, the β-Ag_2_MoO_4_ microcrystals synthesized by the coprecipitation method achieved the highest photocatalytic efficiency, reaching a discoloration rate of 85.12% under UV–C irradiation after 240 min. This enhanced performance was strongly associated with the stabilization of high-energy (112) facets, the formation of [AgO_5_·xV_O_] clusters, and the resulting increase in active sites for radical generation. Particle size was also significantly influenced by the choice of synthesis method. For instance, Xu et al. explored the synthesis of Ag_2_CrO_4_ using microemulsion, coprecipitation, and conventional hydrothermal processes.^62^ The microemulsion method yielded the smallest particles (∼30 nm), which increased the material’s surface area and enhanced photocatalytic efficiency for methylene blue degradation under visible light, reaching a kinetic rate constant (k) of 0.033 min^–1^. These findings underscore how different synthetic strategies can modulate defect density, morphology, and surface properties, ultimately tailoring the performance of Ag-based bimetallic semiconductors for specific applications.

When discussing the synthesis of Ag-based bimetallic semiconductors, techniques such as coprecipitation, solvothermal, microwave-assisted, and sonochemical methods are employed as previously observed. Coprecipitation is widely recognized for its simplicity, low cost, and ease of scale-up, although it may offer limited control over particle size and morphology.^89^ Solvothermal synthesis, on the other hand, allows for better crystallinity and morphological control, but often requires high temperatures, long reaction times, and organic solvents, which may raise environmental concerns.^90,91^ Microwave-assisted and sonochemical methods have attracted attention due to their ability to significantly reduce reaction time and improve energy efficiency.^92–95^ The sonochemical route, in particular, benefits from the generation of localized high temperatures and pressures through acoustic cavitation, leading to the formation of nanostructured materials with unique properties.^96^ However, both microwave and sonochemical approaches still face challenges regarding scalability and uniformity in large-scale production. In practical terms, cost, efficiency, and environmental impact are strongly dependent on the degree of morphological control required for a specific application, making it difficult to define a single ″best″ method. Instead, the selection of the synthesis route should consider not only the targeted physicochemical features of the material but also the feasibility of scaling up the process while maintaining performance and sustainability.

Beyond particle formation and morphological control, these synthesis methods also play a critical role in the modulation of defect density and distribution, which are central to surface and defect engineering strategies in Ag-based semiconductors. The ability to tailor defects, such as vacancies, dislocations, and local distortions, through controlled variations in synthesis parameters has a direct impact on the material’s physicochemical behavior. Different synthetic methods can be tailored to influence structural order, defect distribution, and chemical composition. Moreover, even within a single synthesis route, parameters such as reaction time, temperature, pH, and the presence of surfactants or reductive/oxidative atmospheres can lead to markedly different outcomes in terms of defect formation, crystallinity, and morphology, ultimately modulating the material’s physicochemical behavior. As a consequence, it becomes possible to strategically introduce energy states associated with these defects, tuning the electronic structure and enhancing functional properties relevant to catalytic, electronic, and optical applications.^97^

For example, varying the coprecipitation temperature for synthesizing α-Ag_2_WO_4_ with polyvinylpyrrolidone (PVP) as a surfactant directly impacts the material’s morphology.^63,64^ Higher temperatures promote the longitudinal growth of nanorods, reduce their cross-sectional areas, and introduce surface irregularities, thereby increasing the material’s surface area. The sample synthesized at 30 °C was more effective for methylene blue degradation, whereas the one obtained at 90 °C showed superior photocatalytic performance for methyl orange degradation.^63^ These changes influence the ROS generation, leading to modified photocatalytic responses. Similarly, increasing the temperature in the microwave-assisted hydrothermal synthesis of α-Ag_2_WO_4_ reduces particle size, enhancing photocatalytic efficiency for rhodamine B and rhodamine 6G degradation, and the antibacterial activity of the samples against *E. coli*.^65^ Our research group demonstrated that synthesizing β-Ag_2_MoO_4_ at different temperatures (100–160 °C) via microwave-assisted hydrothermal methods alters the morphology of particles from potato-like to coral-like structures, significantly improving photocatalytic activity against RhB, with the sample synthesized at 120 °C being the most effective, reaching a k of 0.0257 min^–1^, likely due to the participation of crystallite defects, deformations in [AgO_6_] and [MoO_4_] clusters, and the presence of oxygen vacancies throughout the crystal lattice.^66^ Furthermore, calcination temperature also plays a pivotal role, as evidenced by Wang et al., who observed that β-Ag_2_MoO_4_ calcined at 500 °C exhibited superior ammonia detection performance compared to samples calcined at other temperatures.^67^

In addition to temperature, reaction time and pressure also influence defect density and material properties. Prolonged reaction times often yield more ordered materials, while shorter durations can introduce disorder and heterogeneity. High-pressure conditions suppress bulk defect formation, whereas ambient or subatmospheric pressures favor the creation of less dense structures rich in vacancies. For instance, our research group demonstrated that longer irradiation times during microwave-assisted synthesis α-Ag_2_WO_4_ increase the dimensions of hexagonal rods and reduce defect densities, as indicated by the decrease of the PL emission.^68^ These subtle differences among the (001), (010), and (101) surfaces illustrate the strong influence of surface type on material reactivity (see Figure 5). By controlling the combination of these surface terminations within the overall morphology, it is possible to finely tune the distribution and accessibility of active sites, an effect reflected in the enhanced photocatalytic degradation of rhodamine B and antimicrobial performance, particularly in samples synthesized within just 4 min, which exhibited the most effective behavior. In addition, we investigated β-Ag_2_MoO_4_:Eu materials and observed that longer microwave irradiation times led to a gradual reduction in defect density, as evidenced by decreased PL intensity and supported by PALS, which revealed a systematic decrease in positron lifetimes (τ_1_ and τ_2_) and an increase in I_2_, indicating a reduction in defect size and a redistribution in defect types at the nanoscale.^98^ Similarly, Miclau et al. synthesized Ag_2_CrO_4_ under pressures ranging from 400 to 800 bar, observing a morphological transition from irregular polyhedral to rod-like structures at higher pressures.^69^ This transformation directly improved photocatalytic activity for the degradation of methyl orange and methylene blue dyes.

**Figure 5:**
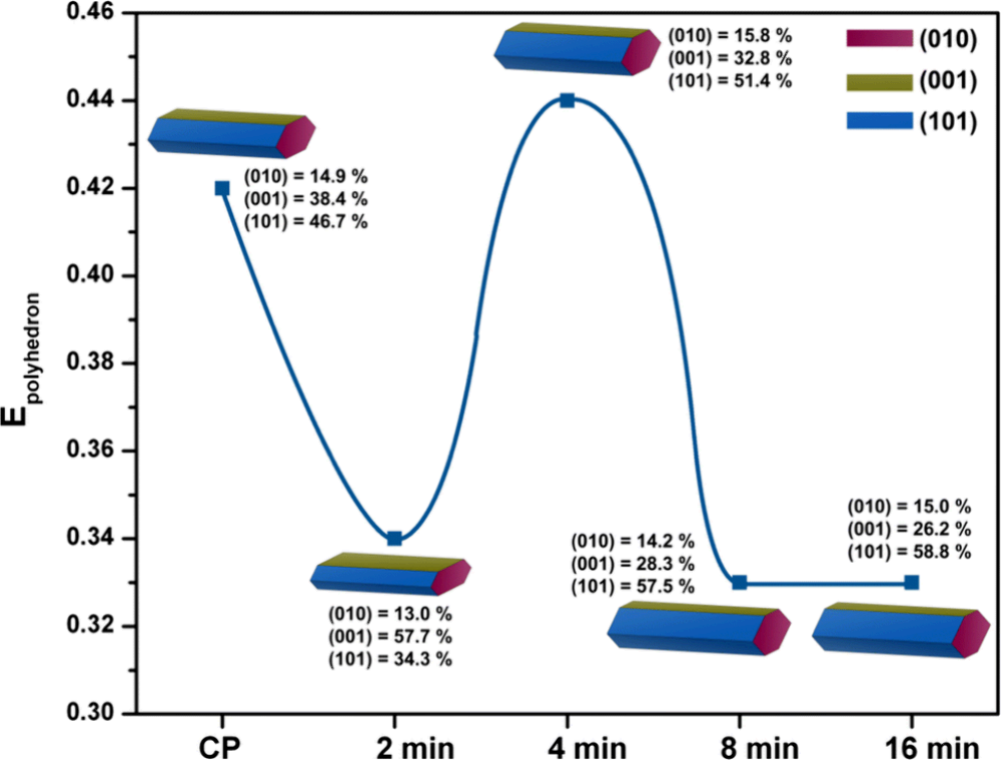
Polyhedron energy profiles and the morphologies for the α-Ag_2_WO_4_ samples synthesized by the CP method followed by microwave irradiation for different times. Reprinted with permission from ref 78. Copyright (2020) Springer Nature.

Beyond physical parameters, chemical factors also play a critical role in controlling defect density in materials. Chemical variables in the synthesis process, such as the use of surfactants (anionic or cationic), reagent ratios (stoichiometric or nonstoichiometric), solvent selection (altering polarity), pH control, and doping with heteroatoms, offer numerous possibilities for modifications during synthesis, enabling precise adjustments to the structural and consequently electronic properties of these materials. These chemical synthesis parameters complement the physical conditions of synthesis, such as temperature, pressure, and reaction time, resulting in a multifaceted approach to defect engineering. For instance, Warmuth et al. demonstrated that simultaneous variation of temperature (0–80 °C) and solvent composition (ethylene glycol and DMSO–water mixtures) can significantly influence the morphology of β-Ag_2_MoO_4_, yielding structures such as spheres, cubes, octahedra, and truncated octahedra depending on the specific conditions applied.^70^ Among these, the truncated octahedra β-Ag_2_MoO_4_ particles exhibited the highest photocatalytic activity under simulated sunlight, likely due to the predominance of (111) crystal facets, which were shown to be more catalytically active than the (100) facets.

Surfactants play a crucial role in modifying surface and bulk defects by selectively interacting with specific crystal facets during the synthesis process. Acting as capping agents, they stabilize high-energy surfaces, influencing nucleation and growth processes.^99^ This interaction often results in the formation of surface vacancies, steps, and other defects that are beneficial for catalytic and adsorption applications. The type of surfactant, cationic, anionic, or nonionic, further modulates electrostatic and steric interactions, thereby affecting the density, nature, and distribution of defects.

Our research group synthesized α-Ag_2_WO_4_ via coprecipitation with and without the anionic surfactant sodium dodecyl sulfate (SDS).^71^ The presence of the surfactant induced a morphological transformation from the typical hexagonal rod shape to a more defined cubic morphology, a change attributed to the preferential stabilization of specific crystallographic facets. SDS promotes the formation of anisotropic nanostructures by selectively adsorbing onto certain crystal surfaces, thereby directing facet-dependent growth during synthesis. This restructuring was accompanied by the emergence of less defect-rich quantum clusters, reducing the material’s photocatalytic activity. These findings reinforce that the presence of SDS not only guides the formation of the cuboid-like α-Ag_2_WO_4_ morphology through selective surface stabilization, particularly of the (100) and (001) facets, but also plays a critical role in reducing the density of under-coordinated clusters, which, although favorable for structural order, ultimately results in lower photocatalytic performance when compared to the defect-rich rod-like counterpart.

Similarly, we used carboxylic acids as surfactants to reduce particle size of α-Ag_2_WO_4_, enhancing RhB photodegradation efficiency.^72^ Carboxylic acids can act as both surfactants and ligands, introducing oxygen-rich functional groups that affect the chemical environment around defect sites.^100^ This effect is closely linked to the dynamic interplay between the formation of the aqueous Ag complex and the chelation process with the carboxylate groups. The competition between Ag–O (from hydration) and Ag–carboxylic acid bonds determine the availability and controlled release of Ag^+^ ions during synthesis. As a result, carboxylic acids act as templating agents, directing particle nucleation and growth. The stability of the chelate complex formed during synthesis plays a key role: lower chelate stability leads to faster Ag^+^ release and larger particles, while more stable chelation results in smaller, well-defined nanostructures. These molecular-level interactions directly influence the final size and morphology of the as-synthesized α-Ag_2_WO_4_ samples.

Surfactants also stabilize metastable phases, as shown by Wang et al. with β-Ag_2_WO_4_ using poly­(methacrylic acid) (PMAA),^73^ and Andrade Neto et al., who synthesized γ-Ag_2_WO_4_ using PVP.^74^ The use of PVP has been shown to induce significant morphological changes in β-Ag_2_MoO_4_ synthesized via microwave-assisted methods.^75^ Specifically, it transformed the morphology from potato-like to cubic, which was associated with improved photocatalytic activity, as confirmed by PL analyses. When PVP was employed in a coprecipitation method using dropwise addition, the resulting β-Ag_2_MoO_4_ particles exhibited a distinctive butterfly like morphology.^76^ PVP is a nonionic surfactant widely used to control particle size and shape due to its strong affinity for metal ions while PMAA is another anionic polymer capable of tuning surface charge and porosity through pH-responsive behavior.^101,102^

In another study, our research group synthesized β-Ag_2_MoO_4_ at varying temperatures (120–150 °C) using a microwave-assisted hydrothermal method, with and without the complexation of Ag^+^ ions by NH_3_.^77^ NH_3_ serves a dual role, not only as a pH modifier but also as a ligand that complexes Ag^+^ ions, forming [Ag­(NH_3_)_2_]^+^ species that influence the nucleation rate and final crystalline structure. While temperature alone did not significantly alter the materials, the inclusion of NH_3_ as a complexing agent had a profound effect on stabilizing the (111) surface. In contrast, when NH_3_ was not used, the (001) and (011) surfaces were stabilized. Additionally, the use of NH_3_ proved essential in modulating the density of oxygen vacancies within the material. Similar findings were reported by Assis et al., who explored the synthesis of Ag_2_CrO_4_ with and without Ag^+^ complexation by NH_3_.^78^ Their results underscored the pivotal role of both the complexing agent and temperature in controlling the material’s morphology (Figure 6). This, in turn, enabled fine-tuning of its photocatalytic, antimicrobial, and cytotoxic activities. Notably, the samples synthesized with NH_3_ showed a preferential exposure of surfaces rich in undercoordinated Ag atoms and coordinated Cr clusters, such as the (011), (001), and (111) planes. These facets, characterized by lower Ag oxidation states and planar [AgO_6_]-derived geometries with minimal distortion, were strongly correlated with enhanced photocatalytic activity, highlighting the surface-dependent nature of the material’s performance.

**Figure 6:**
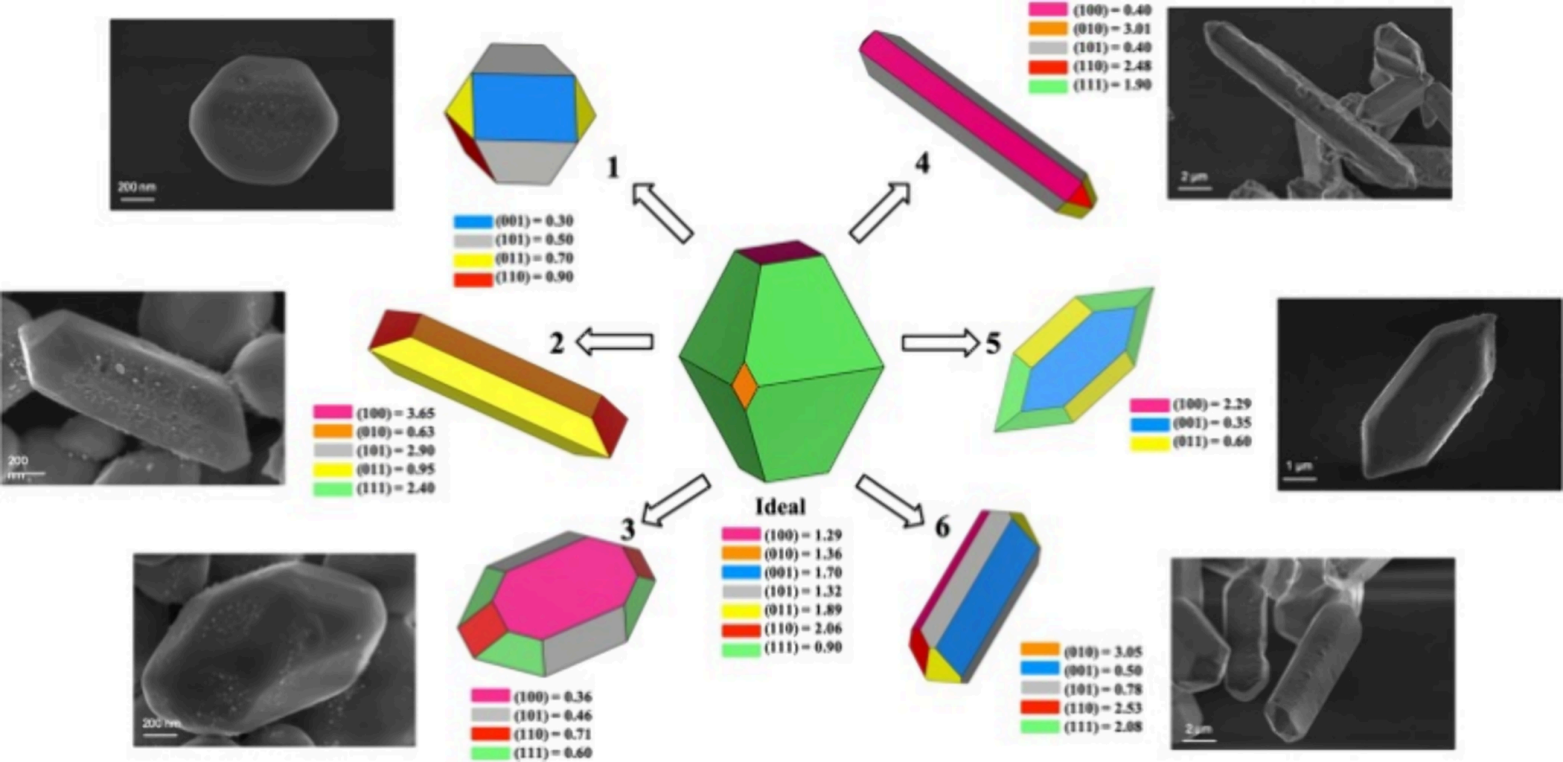
Theoretical and experimental morphologies obtained by tuning the values of *E*_surf_ for Ag_2_CrO_4_. The FE-SEM images for each sample are inserted for comparison purposes. Surface energy value is in J/m^2^. Reprinted with permission from ref 93. Copyright (2021) Springer Nature.

Solvent selection significantly impacts defect formation by affecting reaction kinetics, diffusion rates, and crystal growth.^79^ Polar solvents typically enhance ion dissociation, promoting uniform crystal growth and reducing defect density, whereas nonpolar solvents often slow reactions, leading to disordered or amorphous structures with higher defect densities. Our research group synthesized α-Ag_2_WO_4_ using water, ethanol, and ammonia as solvents via coprecipitation.^80^ Materials synthesized in water and ammonia exhibited flower-like morphologies composed of clustered hexagonal rods, while ethanol yielded dispersed rods with increased oxygen vacancy density. This morphological evolution, confirmed by FE-SEM imaging, is consistent with an oriented aggregation mechanism, where the nature of the solvent influences the self-organization and crystallographic alignment of growing particles. In water, the rapid reaction kinetics favor disordered nucleation, leading to more defective surfaces, while in ammonia, the formation of the [Ag­(NH_3_)_2_]^+^ complex slows Ag^+^ release, allowing more controlled growth and surface definition. In contrast, the use of ethanol restricts particle interaction, promoting the formation of isolated nanorods. This increase in the oxygen vacancies were verified by red-shifted PL emissions, that can enhance the antimicrobial efficiency of the ethanol-synthesized sample.^81^

Similar observations were made for β-Ag_2_MoO_4_, where ethanol stabilized the (001) surface, facilitating ROS production and improving photocatalytic efficiency.^82^ The use of different alcohols in the synthesis of β-Ag_2_MoO_4_ was also evaluated, revealing that increasing the chain length of the alcohol led to noticeable changes in particle size and morphology. Specifically, as the alcohol chain length increased, the morphology shifted from a complex polyhedral structure to the formation of rod-shaped particles.^83^ First-principles calculations combined with experimental analyses showed that this morphological evolution is closely linked to surface exposure, with a higher proportion of the (001) surface correlating with enhanced antibacterial activity, highlighting the critical role of crystal facet engineering in tuning the functional properties of β-Ag_2_MoO_4_.

Reagent ratios also play a critical role in defect engineering, directly impacting vacancy formation. For instance, the excess of metal precursors can enhance the formation of anion vacancies, while deficiencies may lead to cation vacancies. For metastable phases of Ag_2_WO_4_ (β and γ), the molar ratios of reagents are crucial.^23^ Specifically, the stoichiometric ratio of AgNO_3_ to Na_2_WO_4_ governs the formation of distinct cluster species, such as [AgO_6_] and [WO_4_] for γ-Ag_2_WO_4_ at higher concentrations, and [AgO_*x*_] (*x* = 2, 4, 6, 7) and [WO_*y*_] (*y* = 4, 5) for α- and β-Ag_2_WO_4_ at lower concentrations. These variations directly influence the symmetry, order, and morphology of the resulting polymorphs, illustrating the complex interplay between precursor concentration, cluster formation, and crystallization dynamics. Chen and Xu achieved α and β phases through coprecipitation, using stoichiometric amounts for the α phase and a 1:5 Ag/W ratio for the β phase.^84^

The pH of the reaction medium is also a fundamental variable in the synthetic chemical environment and, consequently, in the formation of defects in these materials. Under more acidic conditions, the formation of these materials can be hindered, leading to an increase in defect density and the generation of vacancies. Conversely, in basic conditions, the formation of these materials is often favored, resulting in the development of crystalline phases with a higher degree of order. Thus, even minimal pH adjustments can modify these materials, and this level of control can be valuable for designing materials tailored for specific applications. Della Rocca et al. conducted a statistical factorial design study to synthesize Ag_2_MoO_4_, exploring the effects of surfactant (PVP) addition combined with pH variation in the reaction medium.^85^ Their results demonstrated that pH plays a crucial role in stabilizing the α phase over the β phase of Ag_2_MoO_4_. Furthermore, these pH adjustments influence the formation of other phases, leading to the production of Ag_2_O under basic conditions and MoO_3_ in acidic environments. Similarly, pH is a decisive factor in the synthesis of Ag_2_CrO_4_.^86^ When the pH deviates significantly from 9.5 - whether more acidic or basic - it stabilizes alternative phases of Ag chromates (e.g., AgCrO_2_) or Ag oxides, demonstrating the profound impact of pH on phase stability and material properties.

### Doping

Doping is one of the most precise and targeted strategies for defect engineering, enabling atomic-level modifications at the crystal lattice. By introducing foreign ions into the structure, substitutional or interstitial defects are created, along with localized distortions that significantly alter the material’s electronic and optical properties (see Table 2). For example, doping these materials with transition metals can introduce new electronic states within the bandgap, enhancing properties such as conductivity, photoluminescence, and photocatalytic activity. However, the choice of dopant and its concentration must be carefully optimized to avoid clustering or phase segregation, which can diminish the desired effects.

**Table 2:** Summary of Different Dopings in Ag-Based Semiconductors^a^

material	synthetic method	dopant	concentration (%)	morphology	defect analysis	application	ref
α-Ag_2_WO_4_	microwave hydrothermal	Ni^2+^	0–8	hexagonal rod-like and hexagons	PL		^103^
α-Ag_2_WO_4_	microwave hydrothermal	Mo^6+^	25	hexagonal rod-like	PL	photocatalysis of RhB	^104^
α-Ag_2_WO_4_	coprecipitation	Cu^2+^	0–16	hexagonal rod-like	PL and XPS	antimicrobial	^105^
α-Ag_2_WO_4_	coprecipitation	Zn^2+^	0–25	hexagonal rod-like	PL		^106^
α-Ag_2_WO_4_	coprecipitation	V^4+/5+^	0–4	hexagonal rod-like and parallelepiped-like	PL, XPS, and EPR	sulfide Oxidation	^107^
β- and α-Ag_2_WO_4_	coprecipitation	Eu^3+^	2		PL		^108^
α-Ag_2_WO_4_	coprecipitation	Eu^3+^/Li^+^	1–8	hexagonal rod-like	PL		^109^
α-Ag_2_WO_4_	coprecipitation	Pr^3+^, Sm^3+^, Eu^3+^, Tb^3+^, Dy^3+^ and Tm^3+^	1	hexagonal rod-like	PL		^110^
β-Ag_2_WO_4_	coprecipitation	S^2+^, P^3+^, and B^3+^	20	hexagonal rod-like and irregular shape	XPS	photocatalysis of carmine dye	^111^
β-Ag_2_MoO_4_	coprecipitation	Eu^3+^	0–1	potato-like and cubes	PL		
β-Ag_2_MoO_4_	microwave hydrothermal	Eu^3+^	1	cubes	PL and PALS		^98^
β-Ag_2_MoO_4_	coprecipitation	Dy^3+^	0–2.5		PL and PALS		^112^
Ag_2_CrO_4_	coprecipitation	Eu^3+^	0–1	irregular shape	PL and XPS	photocatalysis of 4-NP and ciprofloxacin	^113^
Ag_2_CrO_4_	coprecipitation	Zn^2+^	1–4	irregular shape	PL and XPS	photocatalysis of RhB and antimicrobial	^30^

a RhB = rhodamine B; 4-NP = 4-nitrophenol.

The doping process involving transition metals like Mo, Ni, Zn, and Cu has been shown to drastically influence the oxygen vacancy density in α-Ag_2_WO_4_, while also inducing morphology changes that depend on dopant concentration.^103–105^ Increased defect density correlates strongly with the addition of dopant clusters, which modify the electronic densities in both the bulk and the surface of the material. This delicate balance between order and disorder introduced by doping is a key factor in altering the photocatalytic responses of these materials. Pereira et al. doped α-Ag_2_WO_4_ with up to 8% Ni^2+^ and observed that increasing Ni^2+^ concentration led to a reduction in the size of the hexagonal rod-like morphology.^103^ This morphological change was attributed to the stabilization of the surface energy of the (001) facet, which initially suppresses crystal growth along the (110) direction, followed by destabilization of the (010) surface. In this context, Ni^2+^ plays a dual role by decreasing the concentration of oxygen vacancies and reducing the overall defect density of the material. When Mo^6+^ is used as a dopant in α-Ag_2_WO_4_, the microwave-assisted hydrothermal synthesis time plays a key role in determining the type of defects formed in the material.^104^ Shorter synthesis times are associated with a higher concentration of deep-level defects, while longer durations tend to promote the formation of shallow defects. These defect profiles directly influence the photocatalytic performance, with deep-level defects, often linked to electronic states near the conduction and valence bands, enhancing the generation of ROS. On the other hand, doping α-Ag_2_WO_4_ with Cu^2+^ increases the material’s defect density, which results in a loss of morphological definition.^105^ However, this structural disorder contributes to enhanced antimicrobial activity, significantly reducing the minimum inhibitory concentration (MIC) from 500 to 31.25 μg/mL for MRSA and from 125 to 15.62 μg/mL for *C. albicans*. A similar result was observed for Zn^2+^ doping, where the loss of morphological definition was accompanied by an increase in oxygen vacancy density (Figure 7).^106^

**Figure 7:**
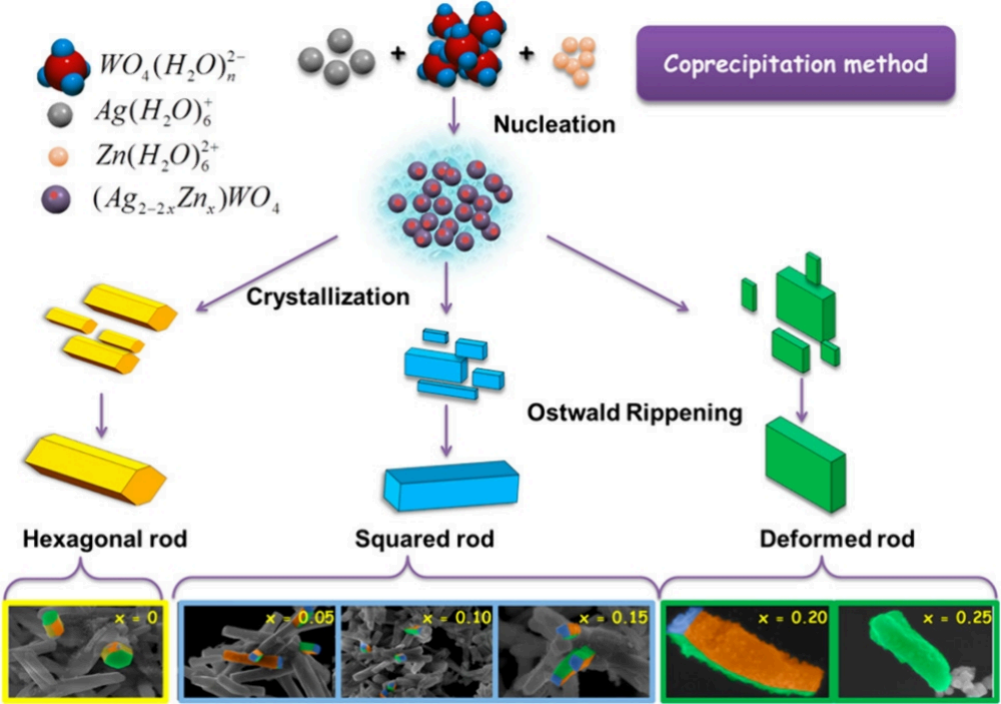
Schematic illustration of the proposed growth mechanism leading to the formation of α-Ag_2–2*x*_Zn*_*x*_* WO_4_ (0 ≤ *x* ≤ 0.25) microcrystals. Reprinted with permission from ref 105. Copyright (2017) American Chemical Society.

For instance, Gupta et al. observed that high concentrations of Eu^3+^ ions could restructure the crystal lattice of α-Ag_2_WO_4_, leading to the formation of the β phase when synthesized via coprecipitation.^108^ This aliovalent doping strategy not only induced an orthorhombic-to-hexagonal phase transition at room temperature, but also significantly influenced the optical properties of the resulting material. These findings demonstrate that defect engineering through rare-earth doping can not only modulate the structural phase but also tailor the electronic and optical responses of Ag-based semiconductors, potentially extending their functionality to applications. On the other hand, our research group reported that lower concentrations of Eu^3+^ and Li^+^ ions increased the defect density in α-Ag_2_WO_4_, enhancing its optical properties.^109^ The presence of Li^+^ as a codopant facilitated a more homogeneous incorporation of Eu^3+^ into the lattice with minimal structural distortion, relieving internal strain and enabling more efficient red-light emission from Eu^3+^ centers. Rare-earth ions (Pr^3+^, Sm^3+^, Eu^3+^, Tb^3+^, Dy^3+^ and Tm^3+^) at concentrations below 1% have also been effective in altering the PL emission characteristics of α-Ag_2_WO_4_, owing to their unique emission signatures.^110^

Nonmetallic dopants such as S, P, and B have also proven highly effective in tuning the crystallinity, phase composition, and defect density of β-Ag_2_WO_4_, leading to significant enhancements in photocatalytic activity.^111^ Using a CTAB-assisted precipitation method, S-, P-, and B-doped Ag_2_WO_4_ materials were synthesized with controlled nanostructures and increased surface hydroxylation. Among these, S-doped Ag_2_WO_4_ exhibited a notably high defect density and amorphous character, particularly in the β-phase, which was linked to superior photocatalytic and adsorption performance. The presence of mesoporosity and N–S bonds, combined with an optimized β-/α-phase ratio, enabled rapid dye adsorption and efficient charge separation. These results reinforce that nonmetallic doping, when combined with surface-active agents, not only modulates defect structures but also enhances the functional performance of Ag-based semiconductors.

We prepared β-Ag_2_WO_4_ samples doped with Eu^3+^ in concentrations ranging from 0.25% to 1.00% and observed a progressive morphological transformation from irregular, potato-like structures to well-defined cubic particles.^114^ This shift is associated with the incorporation of [EuO_6_] distorted octahedra, which locally disrupt the lattice symmetry and induce rearrangements in electronic density. Morphological analyses further revealed that increasing Eu^3+^ content promoted the formation of surface defects, while simultaneously leading to more geometrically defined particles at higher doping levels, highlighting the dual effect of Eu^3+^ in both defect modulation and morphology control.

For Ag_2_CrO_4_, Eu^3+^ doping proved to be a highly effective strategy for enhancing photocatalytic performance, particularly in the degradation of antibiotics and pesticides.^113^ Even at low concentrations (0.25%), Eu^3+^ incorporation modified the oxygen vacancy density and introduced localized structural distortions, as confirmed by XPS and PL analyses. These changes disrupted the short-range symmetry of the lattice, leading to alterations in particle size, morphology, and electronic configuration. Notably, the doped material exhibited reduced charge recombination and improved catalytic efficiency, underscoring the pivotal role of Eu^3+^ in tailoring both structural and functional properties. Zn^2+^ doping in Ag_2_CrO_4_ led to notable improvements in photocatalytic, antibacterial, and antifungal activities, primarily through structural reorganization rather than modification of oxygen vacancy-related defects.^30^ The introduction of Zn^2+^ ions reduced intrinsic disorder and induced changes in particle size and morphology, resulting in enhanced RhB degradation under visible light. These effects became more pronounced with increasing Zn^2+^ content, reaching optimal performance at 2% doping. Both experimental data and DFT-based theoretical calculations revealed that Zn^2+^ doping modulates the electronic and structural order–disorder balance and alters surface energies, highlighting it as an effective strategy to tune material functionality.

The doping process can significantly influence the morphology, stability, and surface reactivity of synthesized materials by promoting the formation of exposed surfaces composed by mor active sites. When combined with key physical parameters, such as temperature, pressure, and reaction time, and chemical variables including pH, precursor concentration, and the presence of surfactants or complexing agents, doping becomes a powerful tool for tailoring defect structures. Despite its potential, this multifactorial approach is often underexplored. A deeper understanding of the interplay between doping and synthesis conditions enables more rational material design, where the degree of order/disorder and defect distribution can be precisely modulated to enhance the functional performance of the final material.

### Postsynthesis Modifications

Postsynthesis modifications can also be useful in altering the structural and electronic properties of these materials, changing their defect densities and electronic characteristics. Techniques such as electron beam irradiation (EBI) and light beam irradiation have proven effective in modulating these properties (see Table 3). EBI can induce drastic changes in the electronic structure of the material, which are not intrinsic to the irradiation technique itself but are particularly dependent on the nature of the modified material^115,116^ Meanwhile, laser or light irradiation can promote atomic migration, surface modifications, or even phase transitions.^117,118^ These methods open up a wide range of possibilities for structural and electronic alterations in these materials, enabling the creation of new functional materials with enhanced performance in various applications.

**Table 3:** Summary of Different Post-synthesis Modifications in Ag-Based Semiconductors^a^

material	synthetic method	postsynthesis modification	parameters	defect analysis	application	ref
α-Ag_2_WO_4_	coprecipitation	EBI	200 kV	XPS		^119^
α-Ag_2_WO_4_	microwave hydrothermal	EBI	30 kV/30 min		antimicrobial	^120^
α-Ag_2_WO_4_	coprecipitation	EBI	20 kV/20 min			^121^
β-Ag_2_MoO_4_	coprecipitation	EBI	5 kV/5 min			^122^
β-Ag_2_MoO_4_	coprecipitation	EBI	5–20 kV/5 min	PL		^123^
β-Ag_2_MoO_4_	coprecipitation	EBI	30 kV/6 min			^14^
α-Ag_2_WO_4_	coprecipitation	EBI	15 kV/5 min		antimicrobial and antitumor	^124^
		femtosecond laser irradiation	800 nm/30 fs/1 kHz/200 mW			
α-Ag_2_WO_4_	microwave hydrothermal	EBI	30 kV/2 min	PL and XPS		^125^
		femtosecond laser irradiation	800 nm/30 fs/1 kHz/200 mW			
α-Ag_2_WO_4_	coprecipitation	EBI	15 kV/5 min	PL, XPS and Magnetic analyses		^56^
		femtosecond laser irradiation	780 nm/150 fs/1 kHz/200 mW			
α-Ag_2_WO_4_	coprecipitation	EBI	30 kV/30 min		antimicrobial	^126^
		femtosecond laser irradiation	800 nm/30 fs/1 kHz/200 mW			
α-Ag_2_WO_4_	coprecipitation	femtosecond laser irradiation	800 nm/30 fs/1 kHz/200 mW		antimicrobial	^24^
α-Ag_2_WO_4_	commercial	laser irradiation	532 nm/10 ns/50 kHz/150 mJ	PL and XPS	photocatalysis of MO	^127^

a MO = methyl orange.

According to results reported in the literature, particularly by our research group, Ag clusters in Ag-based bimetallic semiconductors are particularly susceptible to the EBI process.^119^ This process induces an increase in Ag–O bond distances within the clusters, resulting in the migration of Ag atoms from the internal structure to the surface of the material. Our research group demonstrated this effect by modifying α-Ag_2_WO_4_, synthesized via a microwave method using PVP as a surfactant, through EBI using a scanning electron microscope operating at 30 kV for 30 min.^120^ This modification led to the formation of Ag nanoparticles decorating the surface of the α- Ag_2_WO_4_ particles. The process involved the internal reduction of Ag ions within the material’s framework, causing an electronic restructuring that significantly enhanced its antimicrobial efficiency. Specifically, it reduced the minimum inhibitory concentration against methicillin-resistant *S. aureus* (MRSA) by a factor of 5. Similar results regarding electron irradiation-induced modifications were reported for the metastable β-Ag_2_WO_4_ phase,^121^ complemented by theoretical calculations and modeling.^128^ Comparable findings were also observed both experimentally and theoretically for Ag_2_CrO_4_,^122^ further demonstrating the transformative potential of electron irradiation in tailoring the properties of Ag-based materials.

The modification of Ag-based bimetallic semiconductors using light or laser irradiation can significantly alter their structural, electronic, and optical properties by inducing localized energy absorption and defect generation.^13^ These effects are primarily due to the interaction of high-energy photons with the material, which can trigger different processes. For instance, laser irradiation can create or heal defects by rearranging atoms within the crystal lattice, leading to changes in oxygen vacancy density or the formation of metallic nanoparticles on the surface due to localized reduction.^129^ This is particularly relevant in Ag-based bimetallic semiconductors, where Ag clusters are highly responsive to photon-induced energy. Lin et al. modified commercial α-Ag_2_WO_4_ using a continuous 532 nm laser in solution, successfully reducing its bandgap from 3.22 to 2.78 eV.^127^ This electronic restructuring was attributed to distortions in the [WO_6_] clusters, leading to enhanced photocatalytic activity for methyl orange degradation and improved hydrogen generation efficiency.

In a previous work, it was observed that femtosecond pulsed laser modification was more effective than electron irradiation for producing highly efficient antimicrobial materials.^24^ The localized yet highly energetic effects of femtosecond pulsed lasers (800 nm) caused significant electronic and structural disruptions, making the material up to 64 times more effective against fungi (*Candida albicans*) compared to the unmodified material, and four times more effective than the material modified with electron irradiation.^126^ Similar results were reported by Macedo et al., who used these two techniques to modify different morphologies of α-Ag_2_WO_4_, achieving antimicrobial efficiencies against methicillin-resistant *S. aureus* that were 64 times higher than those of unmodified materials (Figure 8). Moreover, these modifications have shown potential for selective interactions with cancer cells (MB49) over healthy cells (BALB3T3), offering a promising strategy for the development of novel materials with targeted biomedical applications.^124^ In summary, electron and/or laser irradiation provide a facile *in situ* synthesis of Ag nanoclusters for promoting highly reactive based bimetallic semiconductors with multifunctional properties and then applications.

**Figure 8:**
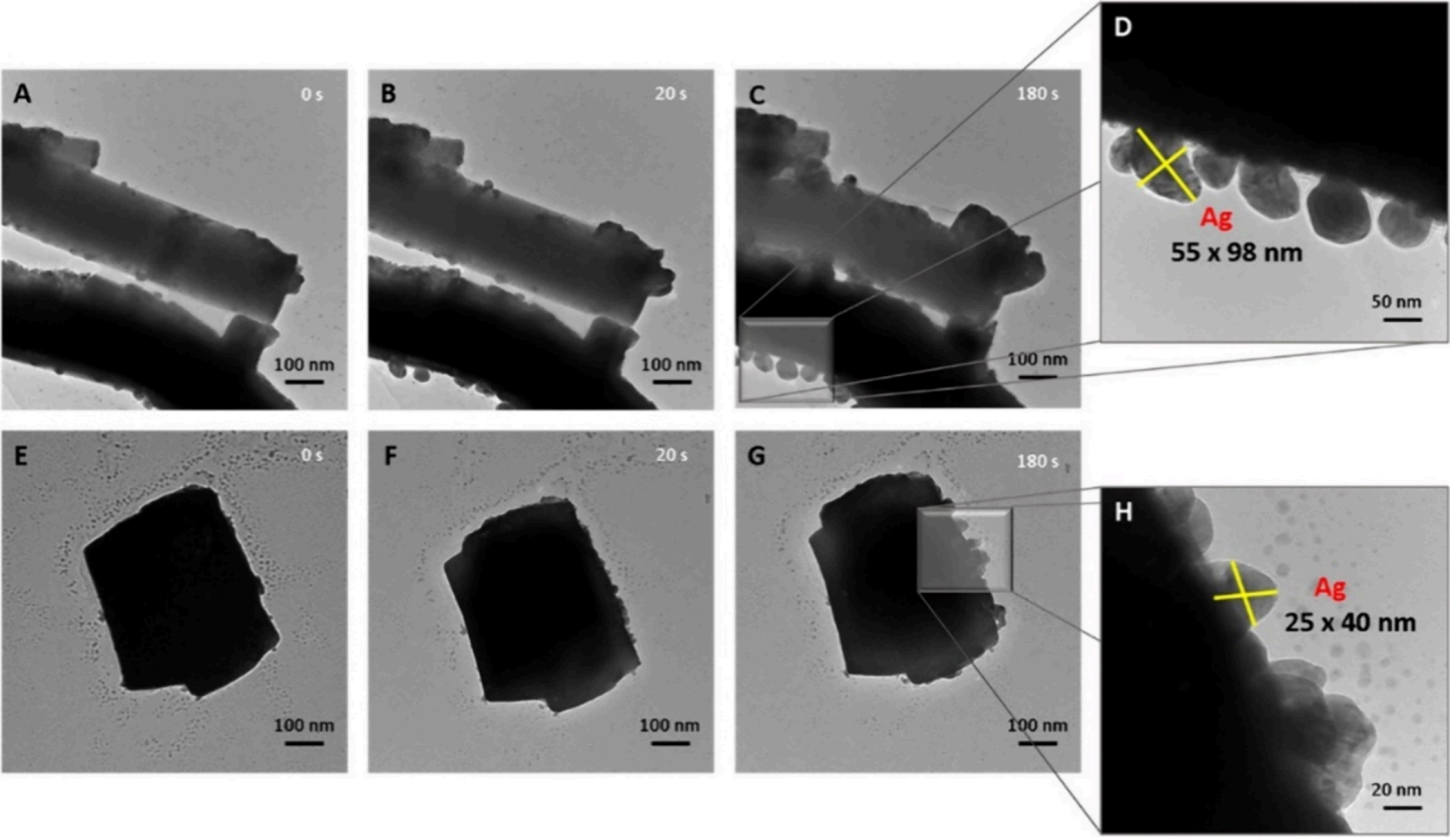
TEM images of Ag NPs/α-Ag_2_WO_4_ composites within a controlled time of exposure to a 200 kV electron beam. (A–D) Ag NPs/α-Ag_2_WO_4_-RE and (E–H) Ag NPs/α-Ag_2_WO_4_–CE samples. (A, E) *t* = 0 s; (B, F) *t* = 20 s; (C, G) *t* = 180 s; (D, H) higher magnifications of the nanoparticles after 180 s. Reprinted with permission from ref 123. Copyright (2019) American Chemical Society.

## Analysis of Defects

The analysis of defects in these materials is crucial, as they significantly influence their electronic, optical, and catalytic properties. Various methods are available to study these defects, with PL spectroscopy being one of the most fundamental techniques. This technique can provide insights into the electronic structure of materials through intracluster interactions, particularly by analyzing the emission spectra they produce.^130,131^ Briefly, when a semiconductor material is excited with a light source (light or laser), electrons are promoted from the VB to the CB with energy above the bandgap value. Through decay processes, these electrons can emit radiation at specific wavelengths. The broad-band theory offers an interpretation of PL emission spectra in terms of defect energy levels within the forbidden region, assessing the medium-range structural order/disorder.^132^ According to this theory, as proposed in the works of Longo et al., higher-intensity emissions in the higher-energy regions of the electromagnetic spectrum (toward the left) correspond to shallow defects. These shallow defects are characterized by energy levels located near the VB and CB and are often associated with the intrinsic structural defects of the material. Conversely, emissions in lower-energy regions (toward the right) correspond to deep defects, which are located further within the forbidden region of the bandgap. Deep defects are frequently associated with oxygen vacancies, and many of these defects may already be occupied by pre-excited electrons, making them distinct from shallow structural defects.^133,134^ In semiconductors, differentiating between shallow and deep defects is critical for understanding medium-range order, as shallow defects can directly influence charge transport, while deep defects can affect electron recombination processes in these materials. Additionally, another important aspect is analyzing the emission intensity of these spectra. Reductions in emission intensity are directly associated with a higher degree of structural organization in the semiconductor, while an increase in intensity is linked to a higher defect density. This makes PL spectroscopy an essential tool for analyzing defects in advanced semiconductor materials. It is also worth noting that PL spectroscopy analyzes the material as a whole, without separating the bulk from the surface, whereas other techniques may have a greater surface-specific character compared to PL.

Our research group has been studying, through PL analysis, how synthetic modifications can alter the defect density of these semiconductors. In a previous study, it was observed that α-Ag_2_WO_4_ synthesized at lower temperatures (100 °C) exhibited a reduction in PL intensity, whereas samples synthesized at higher temperatures (160 °C) showed an increase in PL intensity.^135^ This result demonstrates that temperature can regulate defect density during the microwave-assisted hydrothermal synthesis process. In another work, Lin et al. demonstrated that commercial α-Ag_2_WO_4_ modified with a laser (λ = 532 nm) underwent drastic electronic changes, including a reduction in the bandgap (from 3.22 to 2.78 eV) accompanied by an increase in PL emission intensity, indicating an increase in defect density, particularly in the forbidden region of this material.^127^ On the other hand, the use of the surfactant SDS in the synthesis of α-Ag_2_WO_4_ drastically reduced the PL intensity, suggesting that SDS induces a significant reduction in defect density, accompanied by morphological and surface changes that are critical to the material’s activity.^71^ Teixeira et al. demonstrated that both EBI modification and femtosecond pulsed laser modification increased the density of oxygen vacancies and, consequently, the defect density in α-Ag_2_WO_4_, as shown by PL analysis (see Figure 9).^125^ These findings highlight that the synthesis route is a critical factor in modulating the defect density of these materials, providing a valuable tool for tailoring their properties for specific applications.

**Figure 9:**
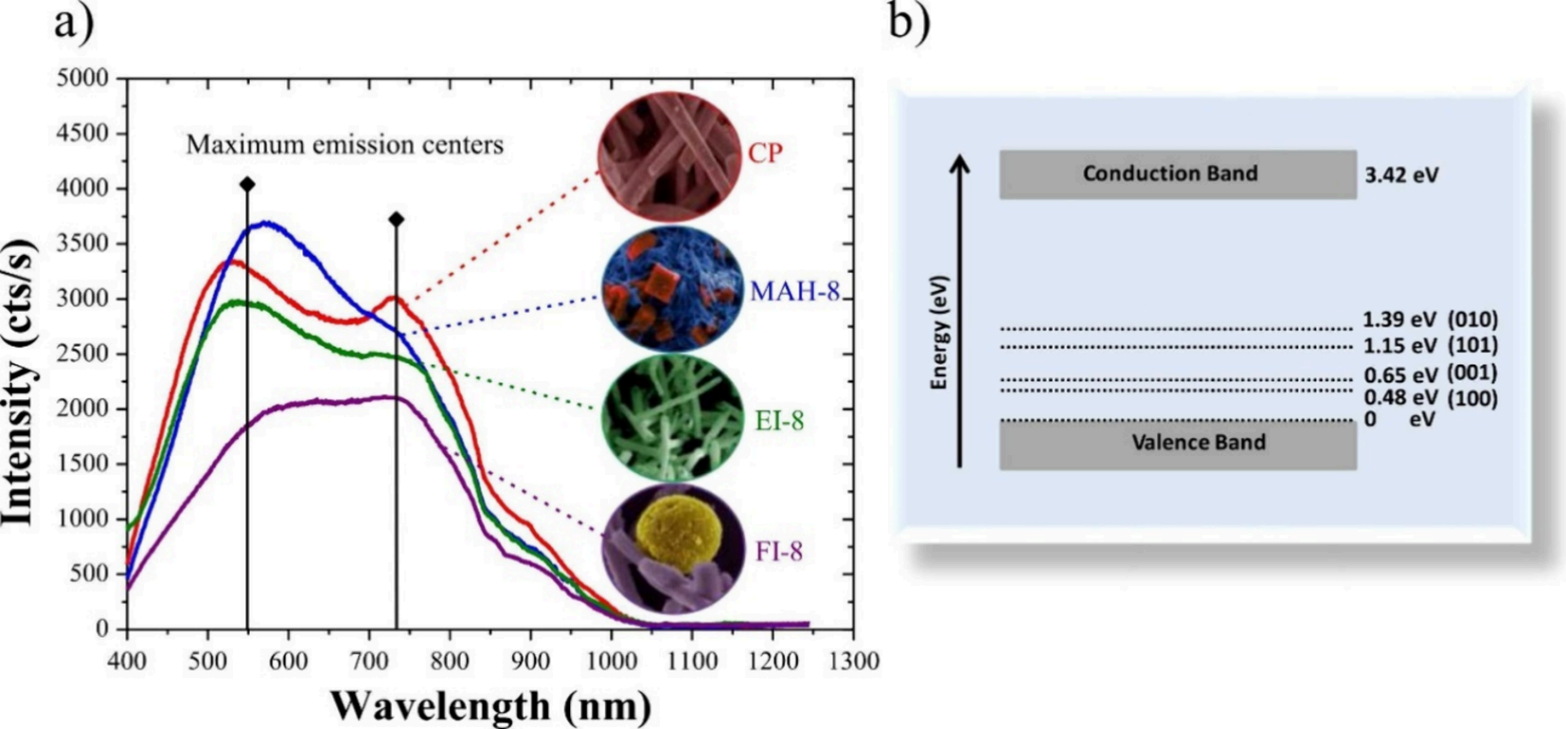
(a) PL spectra and maximum emission centers of the CP, MAH-8, EI-8, and FI-8 samples. (b) Comparative diagram of the band gap value of the optimized structure (3.42 eV) and band gap values for (100), (010), (001), and (101) surfaces. Reprinted with permission from ref 131. Copyright (2022) Elsevier.

Magnetic analyses as a function of applied field and temperature can also play a pivotal role in characterizing defects, particularly the vacancies created in these materials.^44^ These measurements are especially valuable because certain vacancies, such as oxygen vacancies, can exhibit magnetic properties, providing deeper insights into the electronic and structural changes within the material.^136^ Previous studies have shown that variations in local vacancy density are the primary factors influencing the induction of magnetism in α-Ag_2_WO_4_ modified bay electrons and laser.^56^ These findings align with temperature-dependent PL analyses, which offer complementary information about the electronic transitions and defect-related emissions. Magnetic measurements can therefore provide an additional layer of understanding by directly probing the magnetic contributions of defects and correlating them with structural and electronic characteristics. Despite these promising results, further studies are required to establish consistent trends and meaningful associations between vacancy density and magnetic behavior in these materials. By integrating magnetic analysis with PL spectroscopy and other characterization techniques, researchers can develop a more comprehensive understanding of defect dynamics and their impact on the functional properties of Ag-based bimetallic semiconductors.

X-ray photoelectron spectroscopy (XPS) can be a valuable tool for characterizing defects, particularly those related to surface oxygen vacancies, as its analysis typically penetrates between 1–10 nm, equivalent to a few atomic layers of the material.^137^ High-resolution spectra for the O 1s atom can be particularly useful for this analysis. The signal associated with O 1s can be deconvoluted into different components in these materials, primarily corresponding to three binding energy (BE) ranges. These components are associated with lattice oxygen (oxygen atoms constituting the clusters in these materials), oxygen vacancies, and adsorbed oxygen species on the surface (such as hydroxyl groups, adsorbed water, or chemisorbed oxygen).^72^ The asymmetry of the O 1s signal in these spectra arises from the combined presence of these different oxygen species occurring in varying proportions. This makes XPS a powerful technique for detecting and quantifying oxygen vacancies, as well as for understanding their contribution to the electronic structure and surface chemistry of the material. In a previous study by our group, small changes in oxygen vacancies were observed in α-Ag_2_WO_4_ when modified by EBI or laser irradiation.^56^ Ribeiro et al. also demonstrated in their work that the use of carboxylic acids in the synthesis of α-Ag_2_WO_4_ not only alters particle size but also significantly changes the density of oxygen vacancies in these materials, initially reducing them from 49.63% to as low as 28.39% when citric acid was used as a surfactant (Figure 10).^72^ However, it is important to note that inconsistencies exist in the literature regarding the precise identification and quantification of oxygen vacancies using XPS in other semiconductors. Factors such as experimental conditions, data analysis approaches, and specific material properties can lead to misinterpretations of these spectra. Thus, it is of utmost importance to develop a solid and standardized set of methodologies to be applied to the analysis of oxygen vacancies in these and other semiconductors.

**Figure 10:**
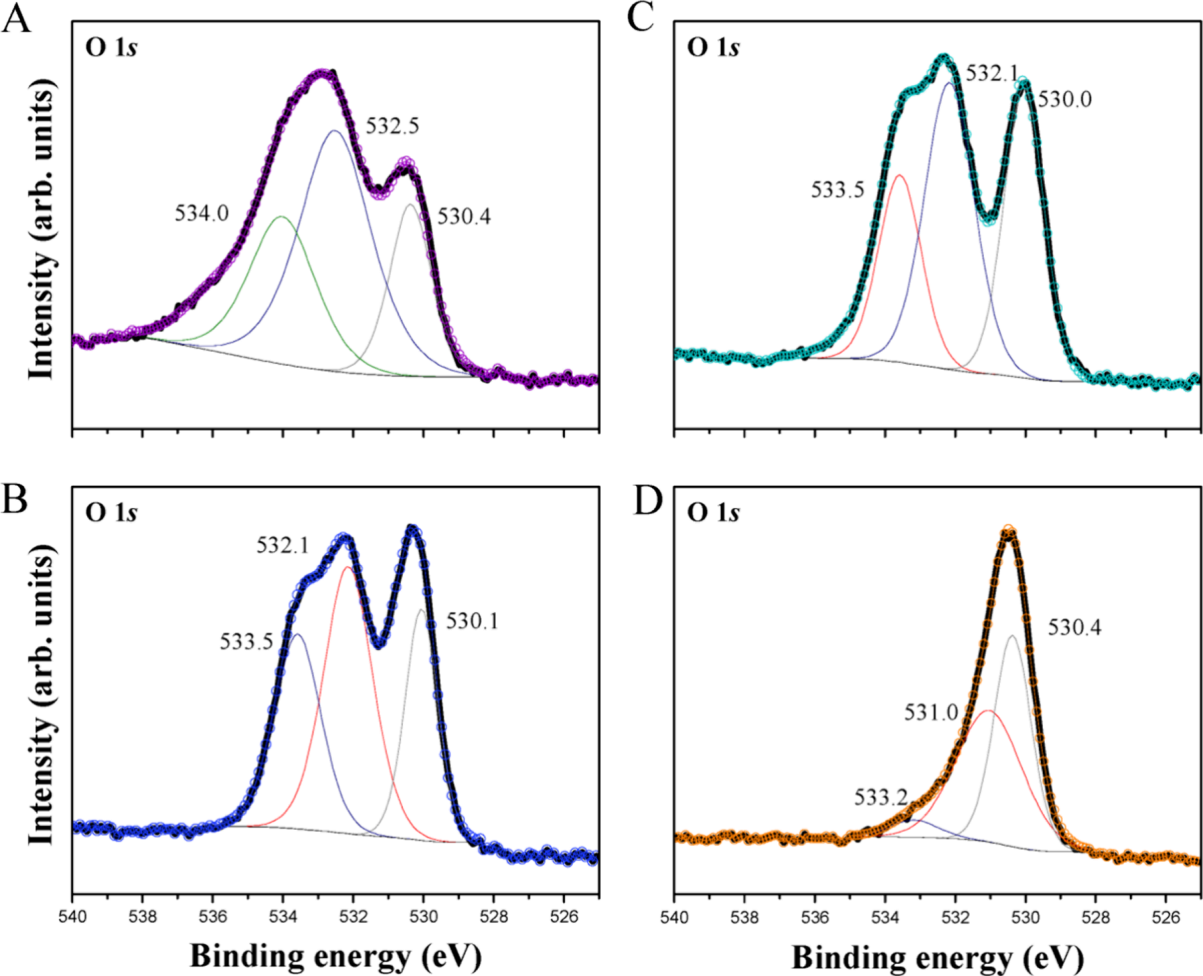
High-resolution XPS spectra of the O 1s orbital of α-Ag_2_WO_4_ (A), α-Ag_2_WO_4_-tartaric acid (B), α-Ag_2_WO_4_-benzoic acid (C), and α-Ag_2_WO_4_-citric acid (D) samples. Reprinted with permission from ref 84. Copyright (2022) MDPI.

Electron paramagnetic resonance (EPR) is a highly effective technique for studying paramagnetic species, such as unpaired electrons associated with defects in semiconductor materials.^138^ Like PL spectroscopy, EPR analyzes the material as a whole, without differentiating between surface and bulk. By detecting distinct magnetic environments, EPR works by measuring the interaction of unpaired electrons with an external magnetic field, providing detailed insights into the electronic structure and defect dynamics of the material. What sets EPR apart from other methods is its unique ability to differentiate between different types of oxygen vacancies when a magnetic field is applied. Since oxygen vacancies can exist in neutral (V_O_), cationic (V_O_·), or doubly ionized (V_O_··) states, cationic vacancies, in particular, can be detected by EPR due to their unpaired electron.^139,140^ Cationic oxygen vacancies produce characteristic signals in the range of *g* = 2.003–2.280, which are widely documented in studies of other semiconductor metal oxides.^141,142^ This precise capability allows EPR to go beyond the mere detection of defects, enabling researchers to identify and characterize their specific types, offering a resolution that other techniques, such as PL and XPS, cannot achieve. In a study by Patrocínio et al., EPR was used to explore the impact of vanadium doping on α-Ag_2_WO_4_.^107^ In addition to clearly identifying the oxidation states of the constituent metals, the results revealed significant changes in the density of cationic oxygen vacancies (*g* = 2.260), with a higher density of these species found in the material doped with 1% vanadium (Figure 11). These findings were consistent with PL and XPS data, confirming the complementary nature of the three methods.

**Figure 11:**
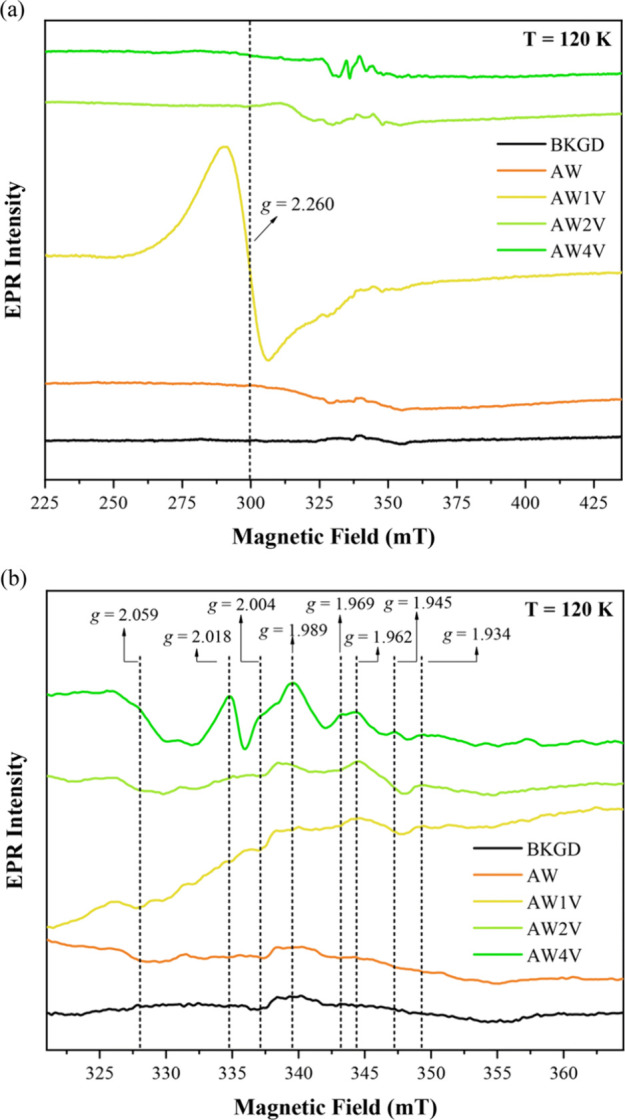
EPR spectra of the undoped (AW) and V-doped (AW1 V, AW2 V, and AW4 V) samples recorded at 120 K. (a) Magnetic field swept from 225 to 435 mT. (b) Magnetic field swept from 320 to 365 mT. The background spectrum (BKGD) was obtained by measuring an empty sample tube. Reprinted with permission from ref 139. Copyright (2023) Royal Society of Chemistry.

Positron annihilation lifetime spectroscopy (PALS) is one of the most sensitive and precise techniques for analyzing surface defects in materials.^143^ PALS works by detecting the interactions of positrons with the material’s structure. These positrons are annihilated based on the size, concentration, and type of defects in the material, making it an extremely useful technique not only for analyzing oxygen vacancies but also for measuring any voids within the material.^144^ This analysis is particularly useful for identifying subtle changes in defect characteristics, such as complex vacancy clusters and their surrounding chemical environments, which other techniques may not detect.^145^ PALS has a greater penetration depth than XPS, typically reaching up to several tens of nanometers, making it highly effective for surface analysis. The penetration depth depends on the positron energy and the density/porosity of the material being analyzed. Our group has demonstrated in several studies the use of PALS to analyze surface defects in Ag-based bimetallic materials (Figure 12). In the case of α-Ag_2_WO_4_, the use of different solvents during synthesis (such as water and ethanol) resulted in a drastic alteration in the nature and size of the surface defects observed.^41^ Samples synthesized in water contained smaller defects, specifically Ag–O vacancy complexes, with an average size equivalent to a trivacancy, formed by two oxygen vacancies surrounding one Ag vacancy. On the other hand, samples synthesized in ethanol exhibited larger and more complex defects, primarily involving Ag vacancies. For this material, it was found that after the EBI process, these defects could be further modified, leading to an increase in the total defect concentration. This indicates that EBI promotes significant changes in the defect environment, increasing both the size and number of these defects. Macchi et al. also employed PALS to investigate how microwave irradiation time influences the size and type of defects in β-Ag_2_MoO_4_:Eu with a fixed doping concentration of 1%.^98^ Their results showed that as the microwave irradiation time increased, a reduction in the positron annihilation lifetime was observed, corresponding to a decrease in the size of these complex defects. This suggests that extended microwave irradiation promotes the stabilization of smaller defect structures, further highlighting the sensitivity of PALS in monitoring subtle defect transformations. Similarly, Sudarshan et al. analyzed the impact of Dy^3+^ doping on β-Ag_2_MoO_4_ obtained via the coprecipitation method at room temperature.^112^ Their results showed that the incorporation of Dy^3+^ ions generates cation vacancies and surface defects, as confirmed by PALS, which also provided evidence of Dy^3+^ stabilization at Ag^+^ sites.

**Figure 12:**
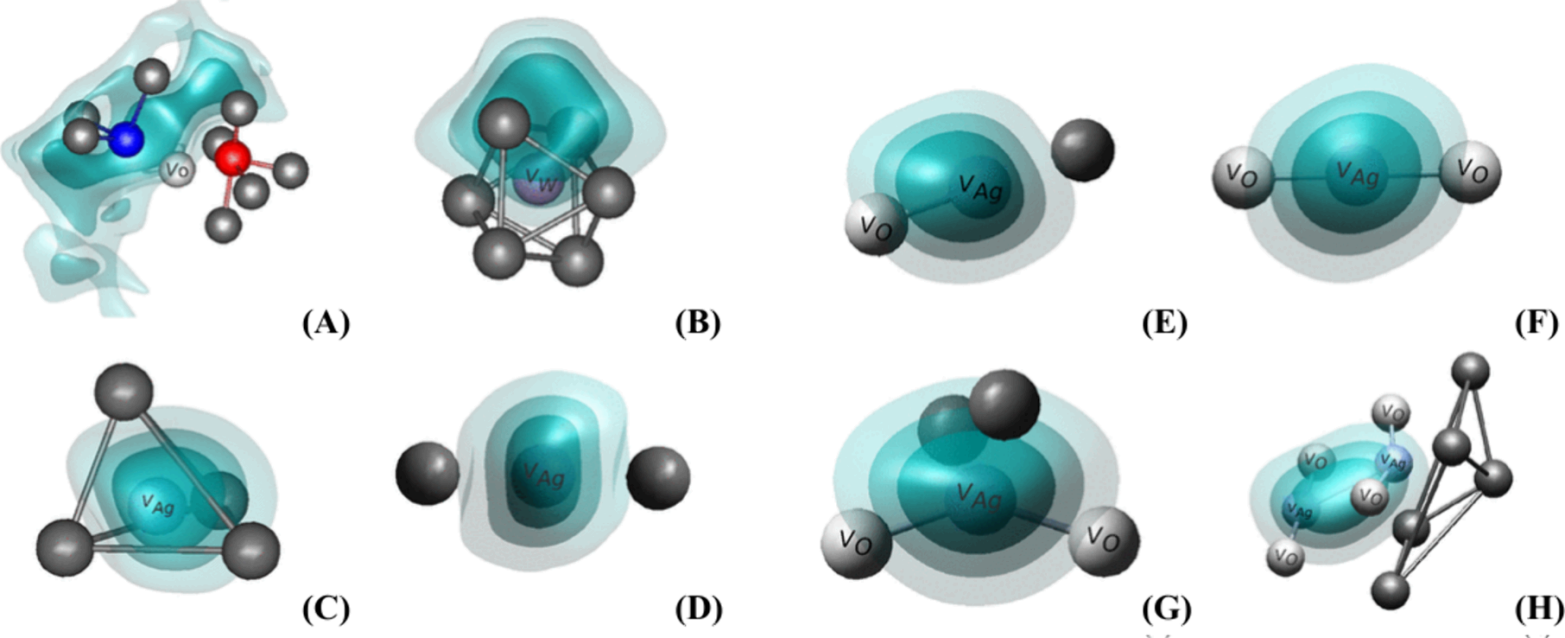
Positron wave function isodensities calculated for monovacancy states: (A) *V*_O_ created between one [WO_6_] and one [AgO_4_] cluster; (B) *V*_W_ created inside a [WO_6_] cluster; (C) *V*_Ag_ formed inside a [AgO_4_] cluster; and (D) *V*_Ag_ created within a [AgO_2_] cluster. The positron isodensities are represented by three surface contours, which correspond to 70, 50, and 30% of the maximum positron density. For the sake of clarity, only the participating clusters are shown in the figures. W atoms are represented in red, Ag atoms in blue, and O atoms in dark gray. Missing atoms related to the different monovacancies are labeled. Positron wave function isodensities calculated for vacancy complexes: (E) *V*_Ag_ + *V*_O_ divacancy created inside a [AgO_2_] cluster; (F) *V*_Ag_ + 2*V*_O_ trivacancy corresponding to the whole missing [AgO_2_] cluster; (G) *V*_Ag_ + 2*V*_O_ trivacancy formed inside a [AgO_4_] cluster; and (H) 2*V*_Ag_ + 4*V*_O_ hexavacancy created between a [AgO_2_] cluster and a neighboring [AgO_7_] cluster. The positron isodensities are represented by three surface contours, which correspond to 70, 50, and 30% of the maximum positron density within the contour. For the sake of clarity, only the participating clusters are shown in the figures. Ag atoms are shown in blue and O atoms in dark gray. Missing atoms related to the different monovacancies forming the vacancy complexes are labeled. Reprinted with permission from ref 139. Copyright (2021) American Chemical Society.

## Trends and Perspectives

The future of Ag-based semiconductors relies increasingly on refined defect engineering strategies to fully exploit their potential across diverse fields such as photocatalysis, antimicrobial treatment, and gas sensing.^49,50,146^ These materials, particularly in their bimetallic and oxide forms (Ag_2_WO_4_, Ag_2_MoO_4_, and Ag_2_CrO_4_), benefit from intrinsic and extrinsic defect structures, including oxygen vacancies, cation deficiencies, and lattice distortions, which can modulate their electronic structure, surface reactivity, and light absorption characteristics. However, unlocking these advantages comes with significant challenges. A major hurdle lies in the reproducible control of defect type, concentration, and spatial distribution during synthesis. Small deviations in conditions such as pH, precursor ratio, or reaction temperature can dramatically alter the defect landscape. As a result, material properties may vary unpredictably, limiting scalability and industrial adoption. Moreover, precise defect tuning at the laboratory scale often proves difficult to replicate in large-scale processes, leading to inconsistencies in performance.

In addition, Ag-based semiconductors suffer from several material-specific limitations that can be addressed through tailored defect engineering. One of the most critical issues is photocorrosion, particularly under prolonged illumination, which leads to structural instability.^147^ Introducing stable defect structures, such as deeply trapped oxygen vacancies or substitutional dopants, can help redistribute local charge density, reducing the oxidation of Ag sites and improving photostability. Similarly, the use of aliovalent doping can stabilize Ag in its lower oxidation state,^148^ minimizing Ag^+^ release and thus mitigating ionic leaching, especially relevant in biological or aqueous environments.

A further challenge is that while surface defects enhance charge separation and ROS formation, an excess of uncontrolled defects often results in increased carrier recombination. To counter this, defect profiles must be tuned precisely in terms of both concentration and spatial distribution.^149^ This can be achieved by combining bottom-up synthesis with *in situ* annealing, redox-controlled atmospheres, or defect-selective surfactants that direct defect formation to targeted facets. In antimicrobial systems, defects are key for ROS-mediated membrane disruption, yet maintaining their activity over time is challenging. Here, dynamic defect structures, such as reversible vacancies or redox-active defect clusters, offer a route to regenerate surface reactivity during use. Moreover, codoping strategies can introduce synergistic defect interactions, where one dopant creates active sites while another stabilizes the structure, enhancing both performance and durability.^150^ Ultimately, the success of Ag-based semiconductors under real operating conditions depends on developing defect architectures that are not only active but also thermodynamically and kinetically stable, enabling long-term function without structural degradation.

In complex architectures like doped or multicomponent systems, defect interactions become more nuanced. While not directly addressed in this study, heterojunctions offer a promising route to improve performance by integrating materials with complementary band alignments.^151^ When combined with controlled defect engineering, such systems can potentially boost charge separation efficiency. However, interfacial defects must be carefully managed to avoid new recombination pathways that could compromise the intended synergistic effects.

To navigate these complex interdependencies, theoretical tools like density functional theory (DFT) and molecular dynamics simulations are increasingly indispensable.^5,21,128,152^ These models allow for the prediction of defect energetics, charge distribution, and band alignment, enabling rational design before experimental synthesis. When integrated with machine learning (ML), these tools can process large data sets to uncover nonobvious correlations between synthesis parameters and material performance. Artificial intelligence further enhances this framework by enabling accelerated material discovery through data-driven prediction of defect-property relationships. This approach reduces reliance on trial-and-error experimentation and helps prioritize promising candidates for synthesis and testing.

Ultimately, addressing these challenges requires interdisciplinary collaboration among chemists, physicists, engineers, and data scientists. From materials design to device integration, a unified approach is needed to ensure that defect-engineered Ag-based semiconductors retain their targeted functionalities in real-world applications. With continued innovation in synthesis, modeling, and characterization, these materials are poised to play a transformative role in next-generation technologies for energy conversion, environmental purification, and healthcare.

## ABBREVIATIONS

PL: photoluminescence spectroscopy
XPS: X-ray photoelectron spectroscopy
EPR: electron paramagnetic resonance
PALS: positron annihilation lifetime spectroscopy
SIA: semiconductor industry association
VB: valence band
CB: conduction band
*E*_gap_: band gap
ROS: reactive oxygen species
PVP: polyvinylpyrrolidone
EBI: electron beam irradiation
SDS: sodium dodecyl sulfate
PMAA: poly­(methacrylic acid)
MRSA: methicillin-resistant *S. aureus*
DFT: density functional theory
ML: machine learning
AI: artificial intelligence
